# An interpretable multimodal model for early prediction of delayed hematoma progression in frontal lobe contusion: a machine learning approach

**DOI:** 10.3389/fnins.2026.1897735

**Published:** 2026-08-12

**Authors:** Guoqing Jiang, Xianglong Liu, Qinghua Zhang, Tao Wang, Chenglei Zhang, Zhanfeng Niu, Shengyu Sun, Hua Sun, Yu Zhao, Liang Wu

**Affiliations:** 1 Department of Neurosurgery, General Hospital of Ningxia Medical University, Yinchuan, China; 2 The First Clinical Medical College, Ningxia Medical University, Yinchuan, China; 3 School of Public Health, Ningxia Key Laboratory of Environmental Factors and Chronic Disease Control, Ningxia Medical University, Yinchuan, China; 4 Department of Neurosurgery, The Second Affiliated Hospital, Hengyang Medical School, University of South China, Hengyang, Hunan, China; 5 Department of Laboratory Medicine, General Hospital of Ningxia Medical University, Yinchuan, Ningxia, China; 6 Department of Perioperative Surgery and Anesthesiology, General Hospital of Ningxia Medical University, Yinchuan, China

**Keywords:** delayed hematoma progression, frontal lobe contusion, machine learning, multimodal models, SHAP

## Abstract

**Background:**

Early identification of delayed hematoma progression (DHP) in patients with frontal lobe contusion remains challenging in emergency settings. This study aimed to develop and externally validate an interpretable multimodal machine-learning model integrating routinely available clinical, laboratory, and CT imaging features to predict DHP.

**Methods:**

This retrospective multicenter study included a development cohort of 799 patients and an external validation cohort of 443 patients. The development cohort was divided into a training set and an internal test set using stratified sampling. Feature selection was performed exclusively within the training set using seven complementary methods. Ten machine-learning algorithms were trained and compared using five-fold cross-validation. Model performance was assessed using AUROC, accuracy, sensitivity, specificity, precision, F1-score, calibration analysis, and decision-curve analysis. SHapley Additive exPlanations (SHAP) was used to interpret the final model.

**Results:**

Ten predictors were selected, including baseline contusion volume, hematoma density-related features, hematoma surface area-to-volume ratio, lymphocyte-to-monocyte ratio, admission Glasgow Coma Scale score, glucose-to-potassium ratio, time to baseline CT, and eosinophil count. The support vector machine (SVM) model showed the highest AUROC point estimate in the internal test set, with an AUROC of 0.801, and achieved an external validation AUROC of 0.724, indicating moderate external discrimination.

**Conclusion:**

We developed an interpretable multimodal model for early prediction of DHP in patients with frontal lobe contusion. The model may assist early risk stratification and clinical monitoring, but further prospective, multicenter, and geographically diverse validation is required before broad clinical implementation.

## 1 Introduction

**Figure 1:**
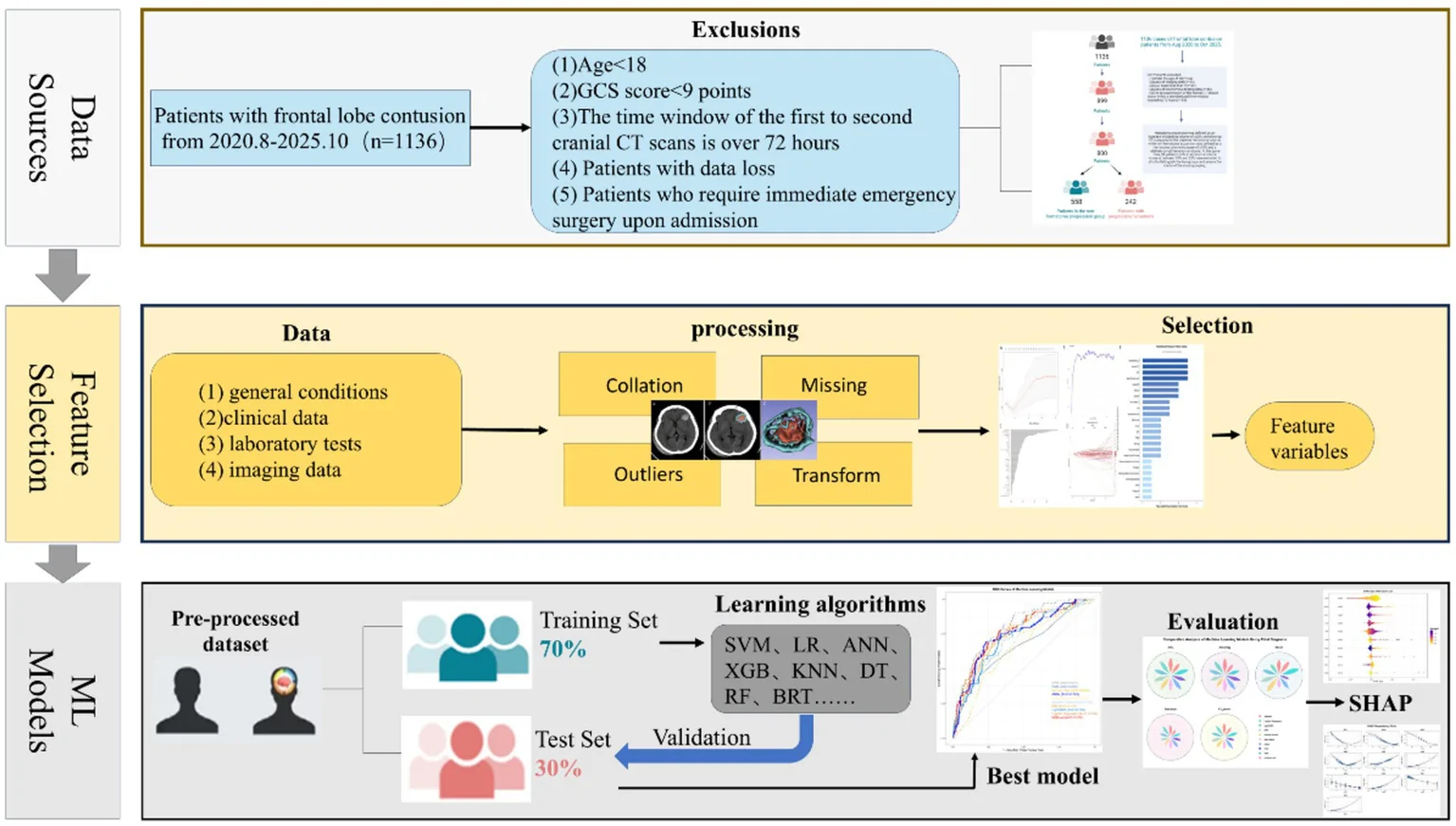
Overall workflow for developing and validating the DHP prediction model.

Traumatic brain injury (TBI) is a leading cause of death and long-term disability worldwide (Adatia et al., 2021; Abebe et al., 2024). As a common and distinct subtype of primary TBI, frontal lobe contusion is frequently complicated by delayed hematoma progression (DHP), which refers to an increase in contusion-related hematoma volume on follow-up CT after the initial injury. In this study, we consistently use the term DHP (Van de Zande et al., 2019; Shafiei et al., 2023). Patients with developing DHP exhibit significantly higher rates of neurological deterioration, emergency surgical needs, and mortality compared to those with contusions in other brain lobes (Zhu, 2022). The incidence of DHP in frontal lobe contusion is reported between 18 and 51%, with most cases evolving within the first 24 h post-injury, while the risk remains substantial for several days (Zhang H. et al., 2024). However, because the early clinical manifestations are often insidious, DHP may remain unrecognized until rapid and potentially life-threatening neurological deterioration occurs. Thus, developing predictive models capable of early identification of patients at high risk for DHP may support early risk stratification, closer monitoring, and timely clinical evaluation. The overall study design and workflow is illustrated in Figure 1.

Currently, most studies on prediction of TBI have focused on spontaneous intracerebral hemorrhage (Zhu, 2022) or generalized cerebral contusions (Zhang H. et al., 2024), with insufficient attention paid to the unique anatomical and clinical features of the frontal lobe. As one of the most vulnerable lobes to traumatic brain injury, the frontal lobe exhibits distinct characteristics that warrant specific investigation, primarily reflected in three aspects. From an anatomical viewpoint, the frontal lobe is vascularized by branches of the anterior and middle cerebral arteries, featuring a dense vascular network with thin vessel walls. Adjacent to the bony structures of the skull base, this region is prone to microvascular tearing following contrecoup injury, which contributes to the formation of delayed hematoma (Van de Zande et al., 2019; Jirlow et al., 2024). In terms of immunoinflammatory responses, the frontal lobe is enriched with immunocompetent cells such as microglia and astrocytes. After trauma, central nervous system (CNS) immune activation in this region is more pronounced, which can readily induce immune dysregulation and exacerbate blood–brain barrier disruption (Wang et al., 2025; Zhang D. et al., 2024). Consequently, the intensity of the inflammatory response in frontal lobe contusions is significantly higher than that in contusions of other brain lobes. Regarding clinical manifestations, patients with frontal lobe contusion often present with mild initial symptoms; however, their neurological function deteriorates more rapidly once DHP occurs (Shafiei et al., 2023), posing a severe threat to patients’ lives. Additionally, most relevant studies have focused on either short-term (2-week) or long-term (>1-month) prognosis, failing to fully capture the rapid early-stage clinical progression characteristics of frontal lobe contusion.

Despite the clinical imperative, robust and validated predictive tools specifically for DHP in frontal lobe contusion with clinical application convenience are still limited. Existing prediction models have predominantly focused on spontaneous intracerebral hemorrhage (Jirlow et al., 2024) or generalized cerebral contusion cohorts (Wang et al., 2025), failing to account for the unique anatomical, immunological, and clinical pathophysiology that characterizes the frontal lobe subtype. This oversight limits their applicability and accuracy for the specific high-risk population of DHP. Furthermore, according to the Transparent Reporting of a multivariable prediction model for Individual Prognosis Or Diagnosis (TRIPOD) and AI statement guidelines (Zhang D. et al., 2024), predictive models must undergo rigorous evaluation across multiple dimensions, including discrimination, calibration, and clinical utility—critical metrics that are frequently underreported in existing literature (Zhang D. et al., 2024; Wang et al., 2022; Zhang et al., 2020; Abujaber et al., 2020; Bark et al., 2024). Recently, the integration of multimodal data has emerged as a powerful approach in biomedical research, offering significant benefits in improving prediction model robustness and cross-scenario applicability (Lee et al., 2024; Li et al., 2022; Morotti et al., 2023). While traditional statistical methods offer interpretability, they are often limited by linear assumptions and an inability to synthesize complex, high-dimensional data from diverse sources (Fletcher-Sandersjöö et al., 2024; Riley et al., 2020). Machine learning techniques present a promising alternative for capturing nonlinear relationships and integrating multimodal features. However, prior applications in TBI have often been constrained by single-modality data, limited generalizability, or poor interpretability (“black-box” problems), resulting in suboptimal performance when applied to specific clinical sub-populations like frontal lobe contusion (Sheng et al., 2022; Zhang et al., 2022). Consequently, the development of predictive models that balance performance, interpretability, clinical feasibility, and generalizability is essential for advancing precision management in frontal lobe contusion.

To address these challenges, this study integrates readily available early clinical data, laboratory biomarkers, and neural radiological imaging features to develop and validate an interpretable, multimodal machine learning model for the prediction of DHP in patients with frontal lobe contusion. The model was developed using data from a single tertiary center and externally validated using an independent cohort from the Second Affiliated Hospital of University of South China. By leveraging explainable machine learning techniques and following rigorous model evaluation, we aimed to develop and externally validate an interpretable multimodal machine-learning model as a potential auxiliary tool for early risk stratification of DHP in patients with frontal lobe contusion. The model was intended to serve as a potential auxiliary tool for early risk stratification and clinical monitoring, rather than as a standalone clinical decision-making system.

## 2 Materials and methods

### 2.1 Research objects and data collection

**Figure 2:**
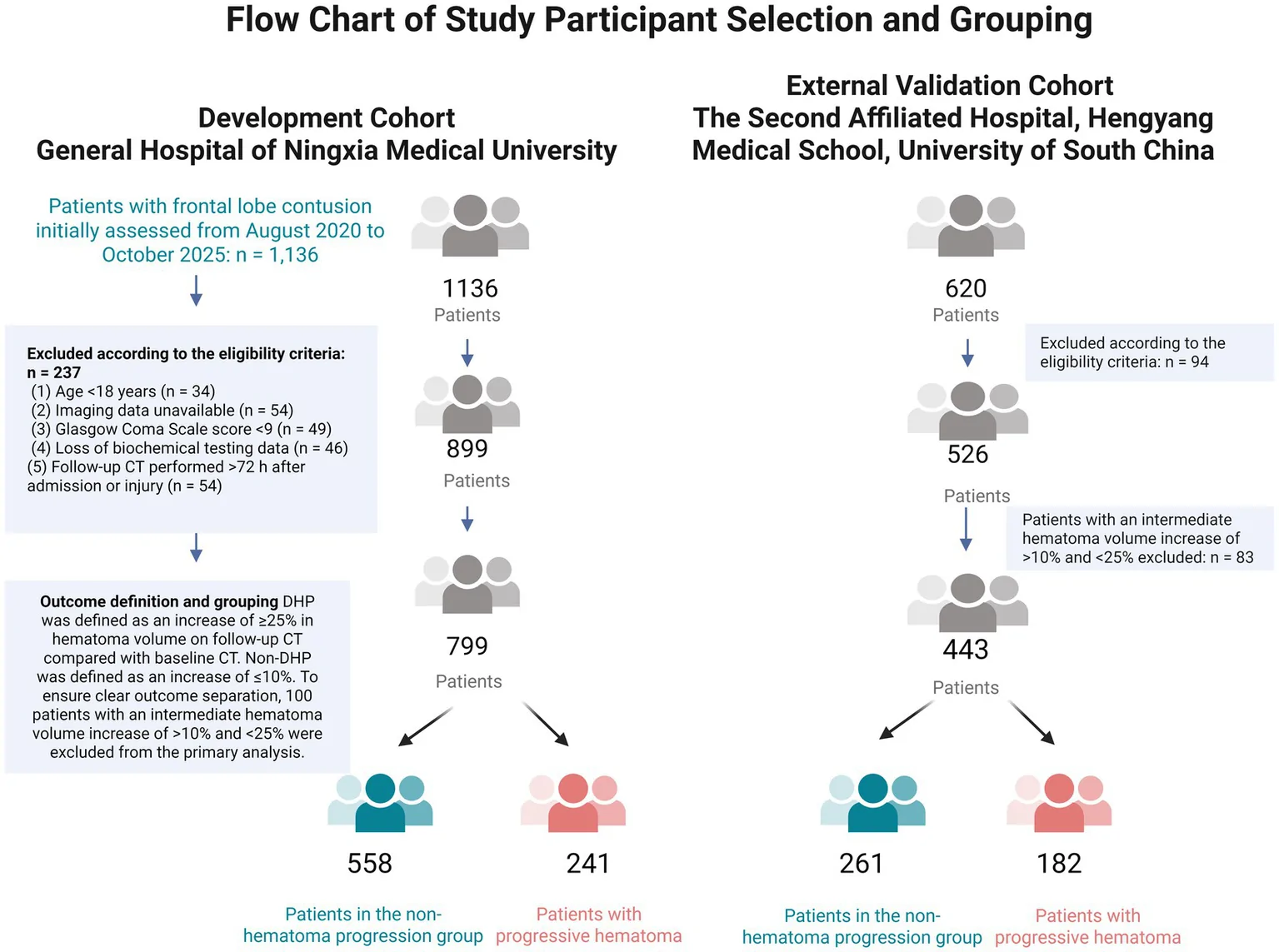
Patient selection flowchart for the development cohort and independent external validation cohort. Excluded patients, DHP cases, and non-DHP cases are shown using distinct colors. The external validation cohort is displayed separately as an independent data source.

This was a multi-center retrospective cohort study including a development cohort and an independent external validation cohort. A total of 1,136 patients with frontal lobe contusion who were hospitalized in the Department of Neurosurgery, Ningxia Medical University General Hospital from August 1, 2020 to October 1, 2025 were initially enrolled. After screening in accordance with the preset inclusion and exclusion criteria, 799 patients were finally included in the final analysis cohort (See the flowchart presented in Figure 2 for more details). The development cohort was randomly divided into a training set (70%, *n* = 560) and an internal test set (30%, *n* = 239) using stratified random sampling to maintain a comparable distribution of DHP status between the two sets. To assess the generalizability of the prediction model, an independent external validation cohort was retrospectively recruited from the Second Affiliated Hospital of University of South China (Hengyang, Hunan Province, China) between January 1, 2020 and December 31, 2025. The same inclusion and exclusion criteria were applied. A total of 620 patients were initially assessed for the external validation cohort. After 94 patients were excluded according to the eligibility criteria and 83 patients with an intermediate hematoma volume increase of >10% and <25% were excluded from the primary analysis, 443 patients remained, including 182 patients with DHP and 261 patients without DHP. This center was selected as the external validation site due to its geographic separation from the development center. However, because both cohorts were derived from Chinese tertiary hospitals, further validation in different geographic regions, hospital levels, and healthcare systems is required before broad generalization. The inclusion criteria were as follows: (1) age ≥ 18 years; (2) admission Glasgow Coma Scale score ≥ 9; and (3) frontal lobe contusion confirmed by baseline cranial CT. The exclusion criteria were as follows: (1) follow-up CT performed more than 72 h after admission/injury; (2) death before follow-up CT; (3) emergency surgery required immediately at admission; and (4) incomplete essential clinical, laboratory, or imaging data.

This study was conducted in strict adherence to medical ethical guidelines. The study protocol was approved by the Ethics Committee of Ningxia Medical University General Hospital (Ethical Approval No.: KYLL-2025-2410). Given the anonymization of all patient data, informed consent was waived for individual participants, which is in compliance with the Declaration of Helsinki and international ethical standards for medical research.

Follow-up CT was defined as the first repeat cranial CT examination performed within 72 h after admission/injury. DHP was determined by comparing hematoma volume between baseline CT and this follow-up CT. DHP was defined as an increase of ≥25% in hematoma volume on follow-up CT compared with baseline CT. In contrast, non-DHP was defined as a hematoma volume increase of ≤ 10% or minimal baseline hematoma volume. To strictly differentiate between the two groups and ensure clear grouping boundaries, 100 patients with a hematoma volume increase ranging from 10% to 25% were excluded from the final analysis. However, because this intermediate group is clinically relevant, these patients were included in a sensitivity analysis to assess the robustness and real-world applicability of the model. Based on these criteria, the 799 enrolled patients were divided into two groups: 558 patients in the non-DHP group and 241 in the DHP group.

Data collection covers four dimensions: demographics and basic clinical indicators, specific clinical data, laboratory tests, and imaging data. All imaging data are taken from the first brain CT image at admission (analyzed through the picture archiving and communication system PACSInfinite3.0, CT scan adopts a unified standard axial thickness of 5 mm). See Supplementary Table S1 for detailed definitions and assignment methods of all variables. The collected data were categorized as follows:

1. Demographics and basic clinical indicators: Age, gender, history of hypertension, history of coronary heart disease, history of diabetes, admission systolic blood pressure, admission diastolic blood pressure, mean arterial pressure, body temperature, and heart rate.
2. Specific clinical data: Admission Glasgow Coma Scale (GCS) score, time from onset to baseline CT, baseline hematoma surface area, baseline hematoma volume, baseline hematoma surface area-to-volume ratio, baseline edema volume, mean hematoma density (HMD), standard deviation of hematoma density (HSD), hematoma density coefficient of variation (HDCV), anterior angle of bilateral lateral ventricles, ultra-early hematoma growth rate, and ultra-early edema progression rate.
3. Laboratory tests: routine blood indicators (white blood cells, neutrophils, lymphocytes, monocytes, eosinophils, basophils, red blood cells, hemoglobin, red blood cell distribution width, platelet count); blood biochemistry indicators (potassium, blood sodium, glucose, urea, creatinine, total bilirubin, uric acid, total protein, albumin, aspartate aminotransferase [formerly referred to as glutamyl-acetic transaminase]); coagulation function indicators (prothrombin time, thrombin time, international normalized ratio, fibrinogen, D-dimer).
4. Imaging data: Presence of subarachnoid hemorrhage, epidural hematoma, subdural hematoma, skull fracture, and bilateral frontal lobe contusion; midline displacement distance; presence of ventricular compression and cerebral sulcus widening/deepening; presence of CT signs including black hole sign, island sign, whirlpool sign, and satellite sign. Additionally, 11 derivative indicators were calculated by combining primary variables in this study, with abbreviations and calculation formulas as follows:

neutrophil-to-lymphocyte ratio (NLR) = neutrophils/lymphocytes;

systemic immune-inflammation index (SIRI) = (neutrophil count × monocyte count)/lymphocyte count;

systemic immune-inflammation score (SII) = (neutrophils × platelets)/lymphocytes;

D-dimer-to-fibrinogen ratio (DFR) = D-dimer/fibrinogen;

platelet-to-lymphocyte ratio (PLR) = platelets/lymphocytes;

monocyte-to-lymphocyte ratio (MLR) = monocytes/lymphocytes;

lymphocyte-to-monocyte ratio (LMR) = lymphocytes/monocytes;

red blood cell distribution width-to-platelet ratio (RPR) = red blood cell distribution width/platelets;

prognostic nutritional index (PNI) = albumin + 5 × lymphocyte count;

aspartate aminotransferase-to-platelet ratio index (APRI) = (100 × aspartate aminotransferase)/platelets;

glucose-to-potassium ratio (GPR) = glucose/potassium.

**Figure 3:**
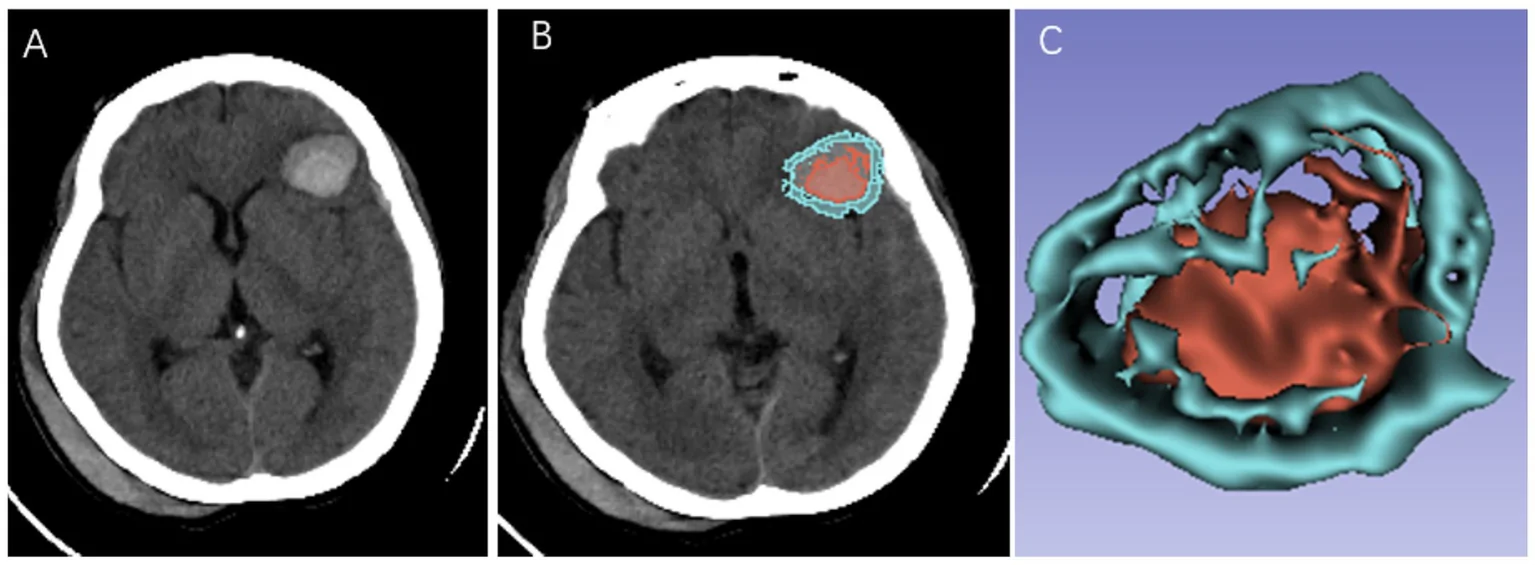
Reconstruction of left frontal lobe contusion and associated edema volume using 3D-Slicer software. **(A)** Left frontal lobe contusion. **(B)** Staining of hematoma and surrounding edema: Red indicates hematoma and green indicates surrounding edema. **(C)** Three-dimensional modeling of hematoma and surrounding edema.

The initial hematoma volume was measured using open-source 3DSlicer software (version 5.8.1). Based on the patient’s first (baseline) and second (follow-up) cranial CT scan images, a fixed Hounsfield unit (HU) threshold (50–100 HU) was used to distinguish hematoma from normal brain tissue (Fletcher-Sandersjöö et al., 2024). The Module function of the software was utilized to create a three-dimensional model of the selected hematoma range and determine the hematoma volume; for patients with multiple frontal lobe contusions, each hematoma volume was measured individually and summed to obtain the total hematoma volume. The measurement and segmentation of all imaging data were independently completed by two professionally trained neuroradiologists (each with ≥ 5 years of experience in brain imaging diagnosis). The two radiologists were blinded to all clinical data of this study, thus adopting a blinded assessment method to effectively avoid subjective expectation bias. When there was a significant divergence in the measurement results between the two radiologists (defined as a difference > 10% for continuous variables or inconsistent judgments for categorical variables), a third senior neurosurgical expert (with ≥ 10 years of experience) conducted a review, and the expert’s judgment was adopted as the final adjudicated value. The cranial CT scan images and 3DSlicer software reconstruction results are presented in Figure 3.

### 2.2 Statistical analysis

To verify the adequacy of the sample size for the prediction model, two methods were employed in accordance with relevant guidelines for prediction model research (Riley et al., 2020): First, based on the rule of thumb that “10–20 events are required per predictor,” the final model in this study included 10 core predictors, thus requiring at least 100–200 DHP events. A total of 241 positive events (DHP) were recorded in this study, which met this requirement. Second, the calculation method proposed by Riley et al. based on the Cox-Snell R-squared value was adopted (Riley et al., 2020), with the following equation: $n=\frac{{\left(z\times \sqrt{2C}\right)}^{2}}{{\left(\mathrm{\Delta p}\right)}^{2}}$ , here z is set to 1.96 (corresponding to α = 0.05), the expected R-squared value ( C ) is set to 0.15, and the acceptable prediction error ( Δp ) is set to 0.05. The minimum number of events required was calculated to be 150. The actual number of positive events (241) in this study far exceeded this standard. In summary, both methods confirmed that the sample size of this study is sufficient to support the construction of a robust prediction model and effectively avoid overfitting.

Missingness was assessed for each candidate predictor. Since all variables had missing rates below 5%, missing continuous variables were imputed using the mean value calculated from the training set, and missing categorical variables were imputed using the mode calculated from the training set. The same imputation parameters were then applied to the internal test set and external validation cohort. Binary variables were coded as 0/1. Multicategory variables, if present, were converted into dummy variables. Continuous variables were standardized using Z-score normalization based on the mean and standard deviation estimated from the training set. The same normalization parameters were applied to the internal test set and external validation cohort.

Additionally, the ratio of the DHP group to the non-DHP group in this study was approximately 1:2.3, indicating a certain degree of class imbalance. We noted that the main clinical goal of this study was to identify patients at high risk of DHP with high sensitivity to avoid missed diagnosis. AUROC was used as the primary threshold-independent discrimination metric, while sensitivity was emphasized as a clinically important screening metric. Specificity, precision, F1-score, calibration, and decision-curve analysis were also evaluated to provide a comprehensive assessment under class imbalance. Given that techniques such as oversampling may introduce overfitting risks and distort the true distribution of features, after comprehensive evaluation, we did not additionally address class imbalance in the final model training. This decision aims to preserve the original distribution characteristics of the data to ensure the generalization performance of the model in real clinical scenarios.

All statistical analyses were performed using R software (version 4.5.1; R Foundation for Statistical Computing, Vienna, Austria). The main R packages utilized included “caret,” “missForest,” “xgboost,” “randomForest,” “e1071,” “KernelSHAP,” “pROC,” “tidyverse,” “lattice,” “ROSE,” “rpart,” “neuralnet,” “ggplot2” and “glmnet.” All *p* values were two-sided, and a p value < 0.05 was considered statistically significant.

### 2.3 Model

#### 2.3.1 Feature screening

Seven mainstream feature selection methods were simultaneously adopted to identify core predictive factors from 74 candidate variables, covering three technical routes: linear regularization, integrated learning, and supervised filtering. The specific parameter settings and implementation details of each method are as follows: ① Least Absolute Shrinkage and Selection Operator (LASSO) regression: Implemented using the “glmnet” package in R. The number of lambda values was set to nlambda = 100, and the regularization parameter alpha = 1 (representing pure L1 regularization). Lambda values were selected through 5-fold cross-validation, and variables with non-zero coefficients were retained; ② Ridge regression: Also implemented via the “glmnet” package, with alpha = 0 (pure L2 regularization). The lambda value was determined based on the lambda.1se criterion (lambda.1se = 20.517), and redundant features were compressed through regularization to avoid overfitting; ③ Elastic-Net (EN) regression: Balancing the advantages of L1 and L2 regularization, with alpha = 0.5. 10-fold cross-validation (nfolds = 10) was used for parameter optimization, and the optimal lambda value was determined as lambda = 0.023; ④ Boosted Regression Trees (BRT): Conducted using the “gbm” package. The key parameters were set as follows: best_iter = 3,450, n.trees = 10,000, interaction.depth = 5, shrinkage = 0.001, and cv.folds = 5. Feature importance was evaluated based on the contribution of each variable to model performance; ⑤ Maximum Relevance Minimum Redundancy (mRMR): Implemented with the “mRMRe” package. The redundancy threshold was set to 0.7, and variables that met two criteria were retained: correlation with the target variable (DHP) ≥ 0.3, and redundancy between features <0.7; ⑥ Extreme Gradient Boosting (XGBoost): Performed using the “xgboost” package. The hyperparameters were configured as eta = 0.1, max_depth = 3, subset = 0.8, colsample_bytree = 0.6, gamma = 0, nrounds = 100, and min_child_weight = 1. Features were ranked based on their gain values, and high-gain variables were prioritized; ⑦ Recursive Feature Elimination (RFE): Implemented using the “caret” and “e1071” packages, with a support vector machine (SVM)-based model as the base classifier. Weakly contributing features were eliminated one by one in each iteration until the model accuracy no longer improved, and the remaining features were retained. The feature importance ranking and coefficient path diagrams obtained from the BRT and Ridge regression methods are presented in Supplementary Figures S1, S2, respectively. Features screened by appearing in at least 3 independent feature-selection algorithms via consensus voting were reserved for final model construction. Per established predictive modeling consensus guidelines, retaining 8–12 predictive covariates strikes a favorable balance between satisfactory discriminative capacity and model parsimony. Our finalized model incorporated exactly 10 predictors within this recommended numerical window, delivering competitive predictive performance with an internal-test AUROC of 0.801 while restraining feature dimensionality to facilitate clinical interpretation and bedside deployment.

#### 2.3.2 Model construction and optimization

The preprocessed data integrated with the screened core features were randomly divided into a training set (70%, *n* = 560) and a test set (30%, *n* = 239) at a 7:3 ratio using stratified random sampling to ensure the consistent distribution of DHP status between the two sets. Using the previously defined training set and internal test set, models were trained in the training set and evaluated in the internal test set. Based on 10 commonly used machine learning algorithms, prediction models were constructed on the training set, including logistic regression (LR), random forest (RF), extreme gradient boosting (XGBoost), light gradient boosting machine (LightGBM), support vector machine (SVM), artificial neural network (ANN), naïve Bayes (NB), k-nearest neighbors (KNN), boosted regression tree (BRT), and decision tree (DT).

Hyperparameter tuning was performed using grid search with five-fold cross-validation. AUROC was used as the primary optimization metric because it provides threshold-independent discrimination assessment. Final model selection also considered sensitivity, specificity, F1-score, calibration, external validation performance, and clinical interpretability. The parameter search ranges and optimal values of the 10 machine learning algorithms are detailed as follows:

1. Support Vector Machine (SVM): Kernel function = radial basis function (RBF); search range of parameter C: [1, 5, 10, 20]; search range of parameter gamma: [0.01, 0.05, 0.1, 0.2]; optimal parameters: C = 5, gamma = 0.01;
2. Extreme Gradient Boosting (XGBoost): search range of max_depth: [2, 3]; search range of nrounds: [75, 100]; search range of colsample_bytree: [0.6, 0.8]; search range of min_child_weight: [1, 3]; search range of eta: [0.01, 0.1, 0.3]; search range of gamma: [0, 0.25]; search range of subsample: [0.5, 0.8]; optimal parameters: eta = 0.1, max_depth = 3, subsample = 0.8, colsample_bytree = 0.6, gamma = 0.25, nrounds = 75, min_child_weight = 3;
3. Logistic Regression (LR): penalty = ‘L2’; search range of parameter C: [0.1, 1, 10]; optimal parameter: C = 1;
4. Light Gradient Boosting Machine (LightGBM): search range of num_leaves: [15, 31, 63]; search range of learning_rate: [0.01, 0.05, 0.1]; search range of n_estimators: [200, 500]; search range of min_child_samples: [5, 10]; fixed parameters: subsample = 0.8, colsample_bytree = 0.8; optimal parameters: num_leaves = 15, learning_rate = 0.05, min_child_samples = 5, subsample = 0.8, colsample_bytree = 0.8;
5. For the parameter search ranges and optimal values of other algorithms, refer to Supplementary Tables S4–S13.

#### 2.3.3 Model evaluation and interpretability

Analysis used the area under the receiver operating characteristic (ROC) curve (AUC), accuracy, sensitivity, specificity and F1 score as performance evaluation indicators (Bo et al., 2023). KernelExplainer is used to calculate SHAP values, and feature effects are analyzed through SHAP swarm plot, dependence plot, and single-sample contribution plot (Lundberg and Lee, 2017). Model discrimination was evaluated using the area under the receiver operating characteristic (ROC) curve (AUC), along with accuracy, sensitivity, specificity, and F1-score (Bo et al., 2023). Model calibration was assessed using calibration curves with the Hosmer–Lemeshow goodness-of-fit test to evaluate the agreement between predicted probabilities and observed outcomes. Decision curve analysis (DCA) was performed to quantify the net clinical benefit of the model across a range of threshold probabilities, comparing the optimal model against the strategies of “treat all” and “treat none,” as well as against other competing models. The DeLong test was used to compare AUC values between different models. The SHAP framework, implemented via the KernelExplainer algorithm, was used to calculate SHAP values for each feature, with feature effects analyzed through SHAP beeswarm plots, dependence plots, and single-sample force plots (Lundberg and Lee, 2017). A completed TRIPOD-AI checklist is provided in Supplementary Table S16.

## 3 Results

### 3.1 Feature screening

**Figure 4:**
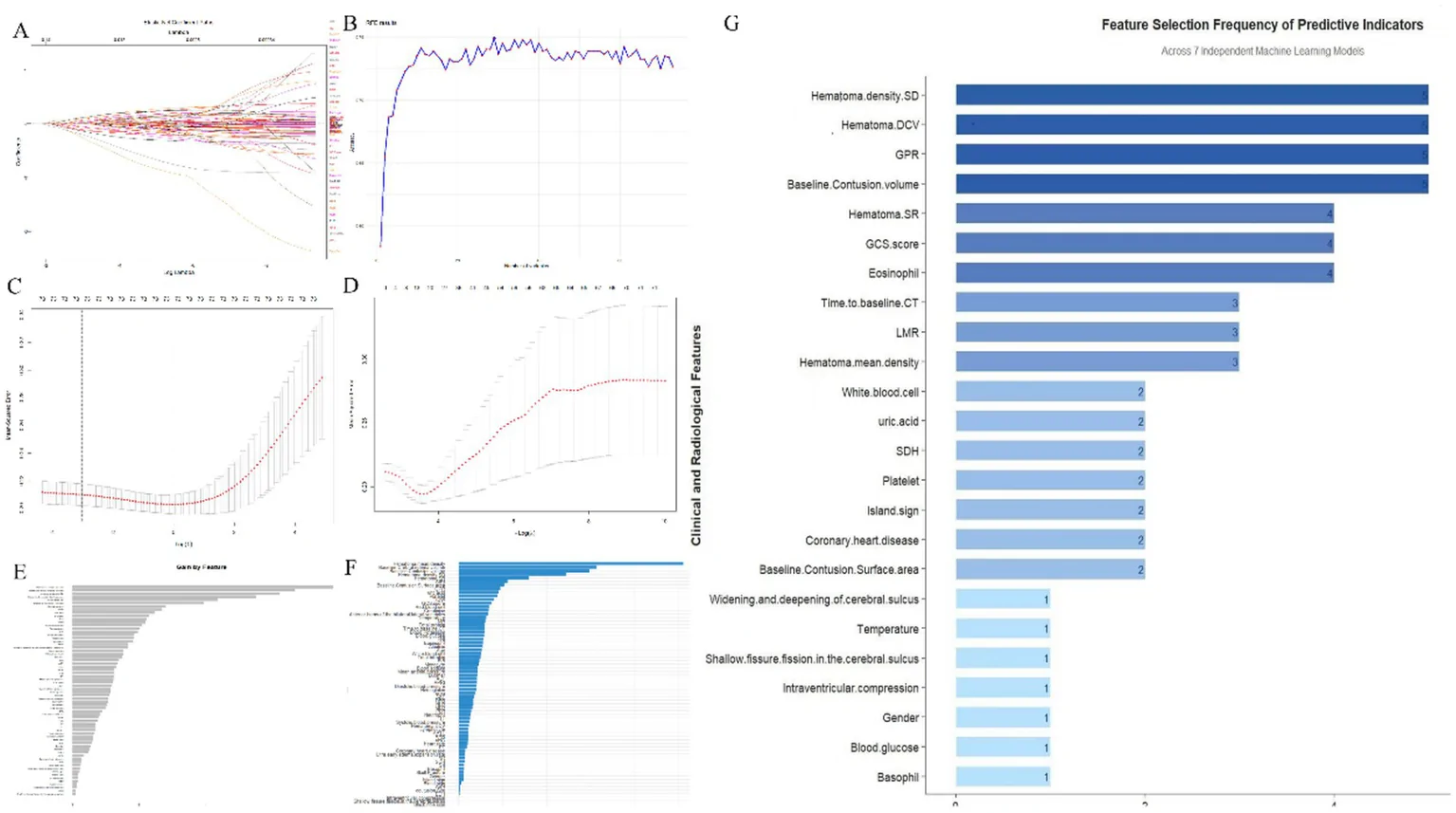
Feature screening results across seven complementary feature-selection pipelines. **(A)** Cross-validation curve of the LASSO regression model for tuning the optimal regularization penalty. **(B)** Performance trajectory from Recursive Feature Elimination (RFE): The line plots predictive accuracy corresponding to feature subsets with varying sizes during iterative feature removal. **(C)** Feature importance ranked by gain metric derived from the XGBoost algorithm. **(D)** Coefficient path plot of Elastic Net regression, illustrating how feature coefficient magnitudes shift alongside regularization strength. **(E)** Horizontal bar chart tallying the selection occurrence of each candidate variable across all screening algorithms. **(F)** Cumulative distribution curve describing the ranked feature selection frequency. **(G)** Horizontal bar plot quantifying cross-method selection frequency for all predictive indicators (screened via 7 independent machine-learning pipelines); bars shaded by frequency strata: dark blue for variables selected ≥4 times (core predictors retained in the final 10-variable model), medium blue for variables selected 2–3 times (intermediate sensitivity-analysis feature pool), pale blue for variables selected only once (low-consensus features reserved for relaxed threshold sensitivity testing).

Seven feature selection methods (including BRT, LASSO, and mRMR) were combined to systematically screen multimodal variables from feature importance, redundancy, and generalization ability. Figure 4 presents representative visualizations of selected feature-selection methods, while complete results from all seven methods are provided in Supplementary Tables S2, S3. Figure 4E summarizes the selection frequency of candidate predictors across all seven methods. For the features screened by each machine learning algorithm, we counted and ranked their screening frequencies. Combined with previous literature reports and clinical expert opinions, 10 core predictive variables were determined, including baseline hematoma volume (BCV), mean hematoma density (HMD), hematoma density standard deviation (HDSD), hematoma density coefficient of variation (HDCV), baseline hematoma surface area-to-volume ratio (HSR), lymphocyte-to-monocyte ratio (LMR), admission GCS score, glucose-to-blood potassium ratio (GPR), time from onset to baseline CT (TBCT), and eosinophil count (EOS). These features not only performed well in algorithmic screening but also held important clinical significance, providing valuable insights for in-depth understanding and interpretation of the model. This ensures that the model in this study not only exhibits high predictive accuracy but also offers substantial value for addressing practical challenges in the field.

Spearman’s rank correlation analysis of the 10 core predictive variables revealed distinct clustered correlation characteristics among variables (Supplementary Figure S3). First, hematoma density-related indicators (HDSD, HMD, HDCV) exhibited strong positive correlations, forming a tight cluster, with the correlation coefficient between HDSD and HDCV reaching as high as 0.90. Second, baseline contusion volume (BCV) showed a strong negative correlation with all density indicators (e.g., correlation coefficient *r* = −0.61 between BCV and HDCV), suggesting the existence of a “hematoma volume-density heterogeneity” pattern. Additionally, a moderate positive correlation was observed between LMR and EOS (*r* = 0.56). The pairwise correlations among the remaining variables were weak (r < 0.3).

### 3.2 Comparison of model performance

**Figure 5:**
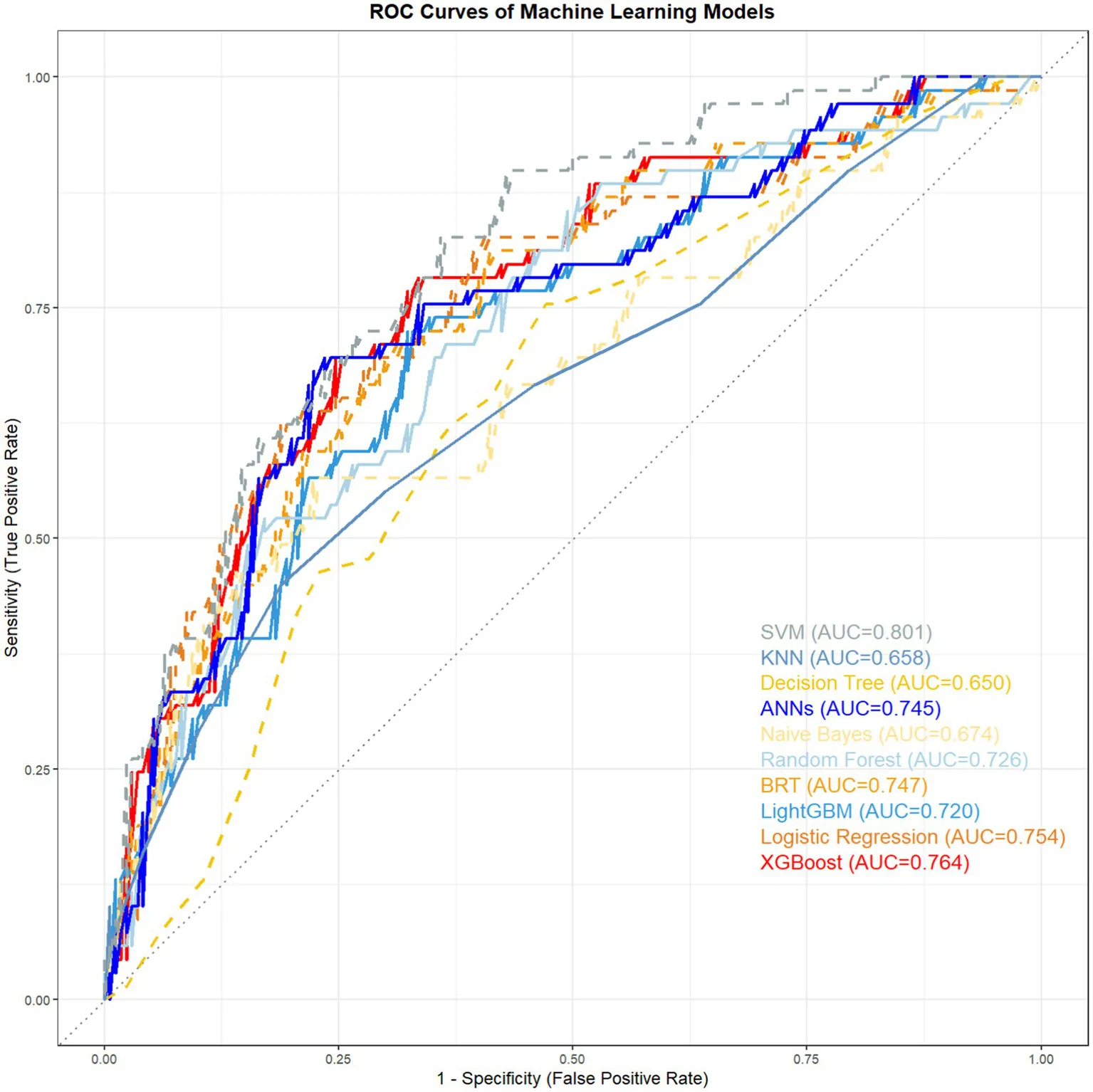
ROC curves of machine-learning models in the internal test set.

**Figure 6:**
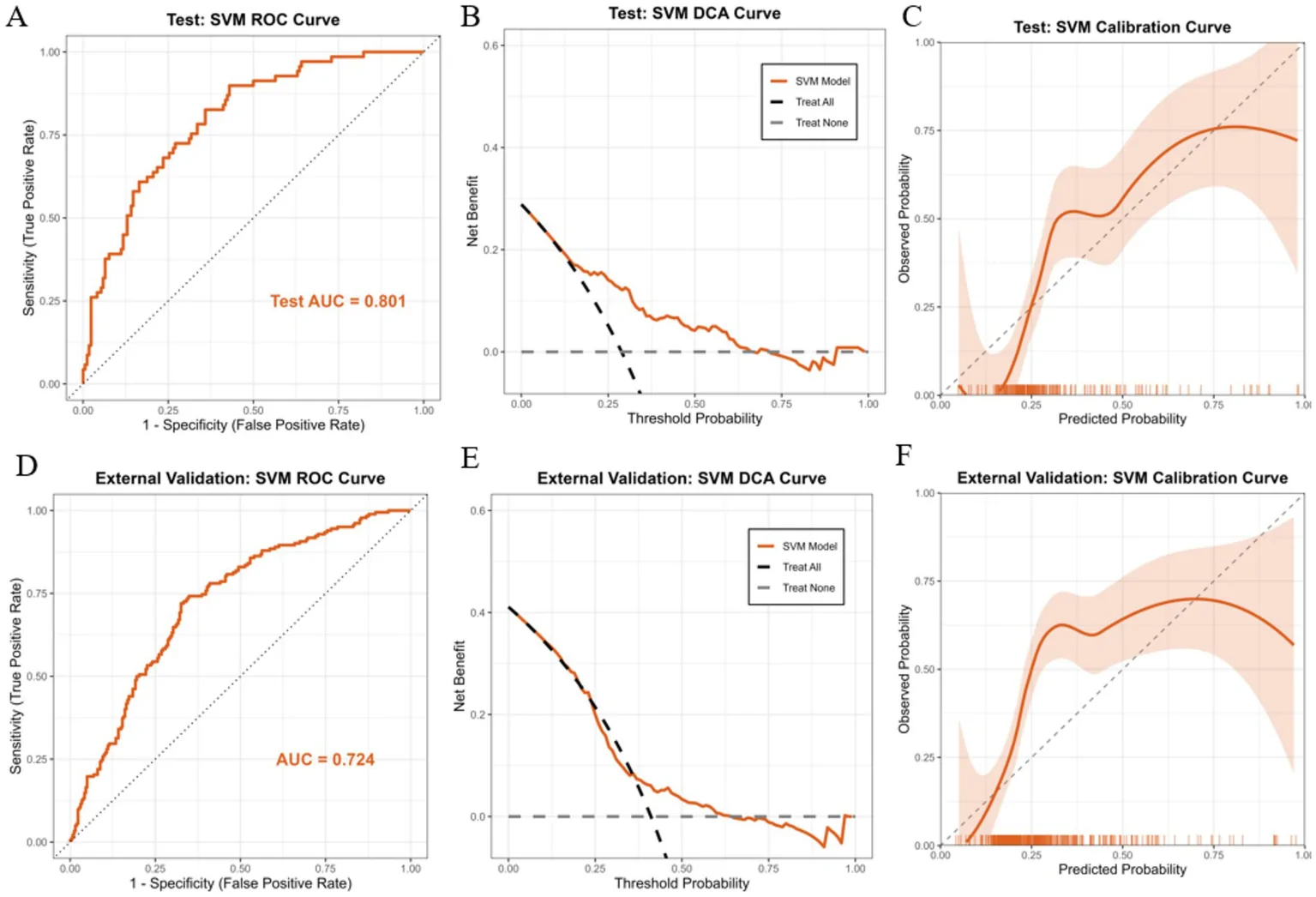
Performance of the final support vector machine model in the internal test set and external validation cohort. **(A)** Receiver operating characteristic curve in the internal test set. **(B)** Decision-curve analysis in the internal test set. **(C)** Calibration curve in the internal test set. **(D)** Receiver operating characteristic curve in the external validation cohort. **(E)** Decision-curve analysis in the external validation cohort. **(F)** Calibration curve in the external validation cohort.

The ROC curves of all models are shown in Figure 5. ROC curve analysis revealed that among the 10 models, the support vector machine (SVM) model exhibited the highest AUROC point estimate (AUC = 0.801, 95% confidence interval [CI]: 0.742–0.859), with a sensitivity of 0.899, specificity of 0.571, F1-score of 0.608, and accuracy of 0.665. Other models also demonstrated certain predictive efficacy, including extreme gradient boosting (XGBoost, AUC = 0.764), logistic regression (LR, AUC = 0.754), and artificial neural networks (ANNs, AUC = 0.745). The ROC curves of all 10 models in the internal test set are presented in Figure 5. Figure 6 presents the ROC, calibration, and decision curves of the final SVM model in the internal test set and external validation cohort. In contrast, the k-nearest neighbor (KNN) model (AUC = 0.658) and decision tree (DT) model (AUC = 0.650) showed relatively lower predictive performance with smaller AUC values.

**Figure 7:**
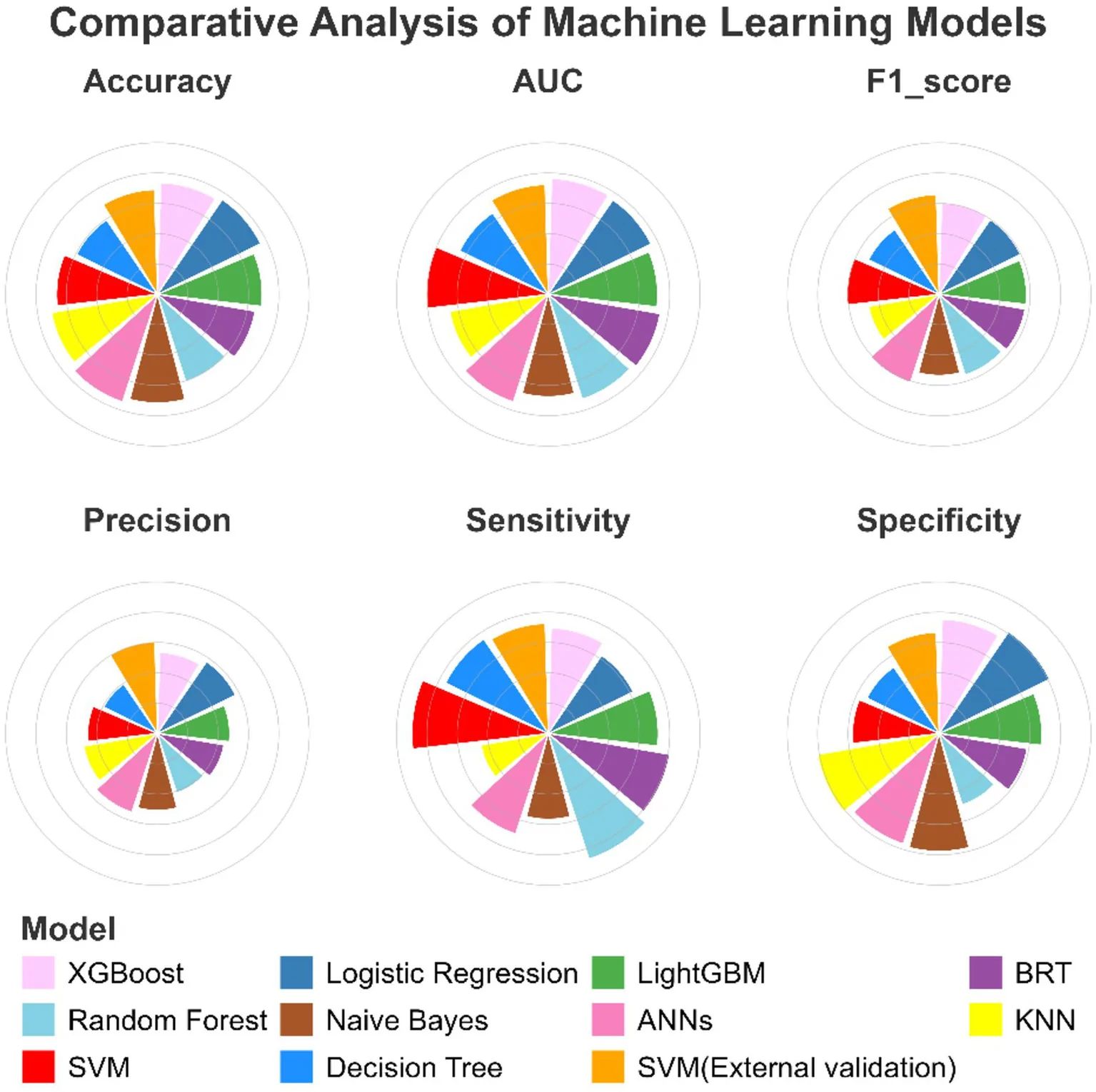
Radial petal chart for multidimensional performance comparison across 10 machine learning algorithms. The radar-style petal plot horizontally benchmarks all included models across six evaluation metrics (accuracy, AUC, F1-score, precision, sensitivity, specificity). Tabular numerical data with full 95% confidence intervals for every model are archived in Supplemental Table S14, while Table 1 in the main text reports the core performance metrics of all 10 models in the internal test set and the external-validation performance of the final SVM model. This visualization addresses the limitation of tabular data: tables only support discrete value lookup, whereas the radial layout enables rapid holistic cross-model comparison of metric balance, intuitively identifying models with skewed performance profiles and validating the robust overall discriminative capacity of the selected SVM framework.

Comprehensive evaluation based on multiple metrics including area under the receiver operating characteristic curve (AUC), accuracy, sensitivity (recall), precision, and F1-score revealed that the support vector machine (SVM) model exhibited the optimal performance in distinguishing between “DHP” and “non-DHP” in frontal lobe contusion. Specifically, the SVM model achieved a sensitivity (recall) of 0.899, suggesting relatively high sensitivity for identifying DHP cases in this dataset. For other models, the XGBoost model yielded an AUC of 0.764, accuracy of 0.736, and F1-score of 0.604; the logistic regression (LR) model showed an AUC of 0.754, accuracy of 0.757, and specificity of 0.812. Models including LightGBM, boosted regression tree (BRT), random forest (RF), and artificial neural networks (ANNs) exhibited varying strengths and weaknesses, whereas models such as k-nearest neighbor (KNN), naive Bayes (NB), and decision tree (DT) demonstrated relatively poor performance across relevant metrics. The performance of each model on the internal test set and the external validation set is summarized in Table 1. More detailed performance metrics of all models are presented in Figure 7 and Supplementary Table S14.

**Table 1:** Performance comparison of 10 machine learning models in the internal test set and external validation cohort.

Data sources	Model name	auc	Accuracy	Sensitivity/recall	Precision	F1 score	Specificity
Test	XGBoost	0.764	0.736	0.696	0.533	0.604	0.753
	Logistic Regression	0.754	0.757	0.623	0.573	0.597	0.812
	LightGBM	0.720	0.690	0.725	0.476	0.575	0.676
	BRT	0.747	0.653	0.812	0.444	0.574	0.588
	Random Forest	0.726	0.603	0.870	0.411	0.558	0.494
	Naive Bayes	0.674	0.716	0.565	0.506	0.534	0.776
	ANNs	0.745	0.745	0.696	0.545	0.611	0.765
	KNN	0.658	0.707	0.449	0.492	0.470	0.812
	SVM	**0.801**	**0.665**	**0.899**	**0.459**	**0.608**	**0.571**
	Decision Tree	0.650	0.594	0.754	0.394	0.517	0.529
External validation	SVM	0.724	0.691	0.725	0.603	0.658	0.667

Bold values indicate the best-performing value for each metric among the 10 machine-learning models in the internal test set.

Additionally, differential analysis of the area under the receiver operating characteristic curve (AUC) among the 10 machine learning models (XGBoost, Logistic Regression, LightGBM, BRT, Random Forest, Naive Bayes, ANNs, Decision Tree, KNN, and SVM) was performed based on the DeLong test *p*-value matrix (Supplementary Table S15). The results revealed that there were significant or extremely significant differences in AUC between multiple pairs of models (*p* < 0.05), including SVM vs. Random Forest (RF), Naive Bayes (NB), Decision Tree (DT), and KNN; and Decision Tree (DT) vs. XGBoost, Logistic Regression (LR), LightGBM, and SVM. In contrast, no statistically significant differences in AUC were observed between BRT and all other models (*p* ≥ 0.05).

### 3.3 Model interpretability

#### 3.3.1 SHAP beeswarm plot

**Figure 8:**
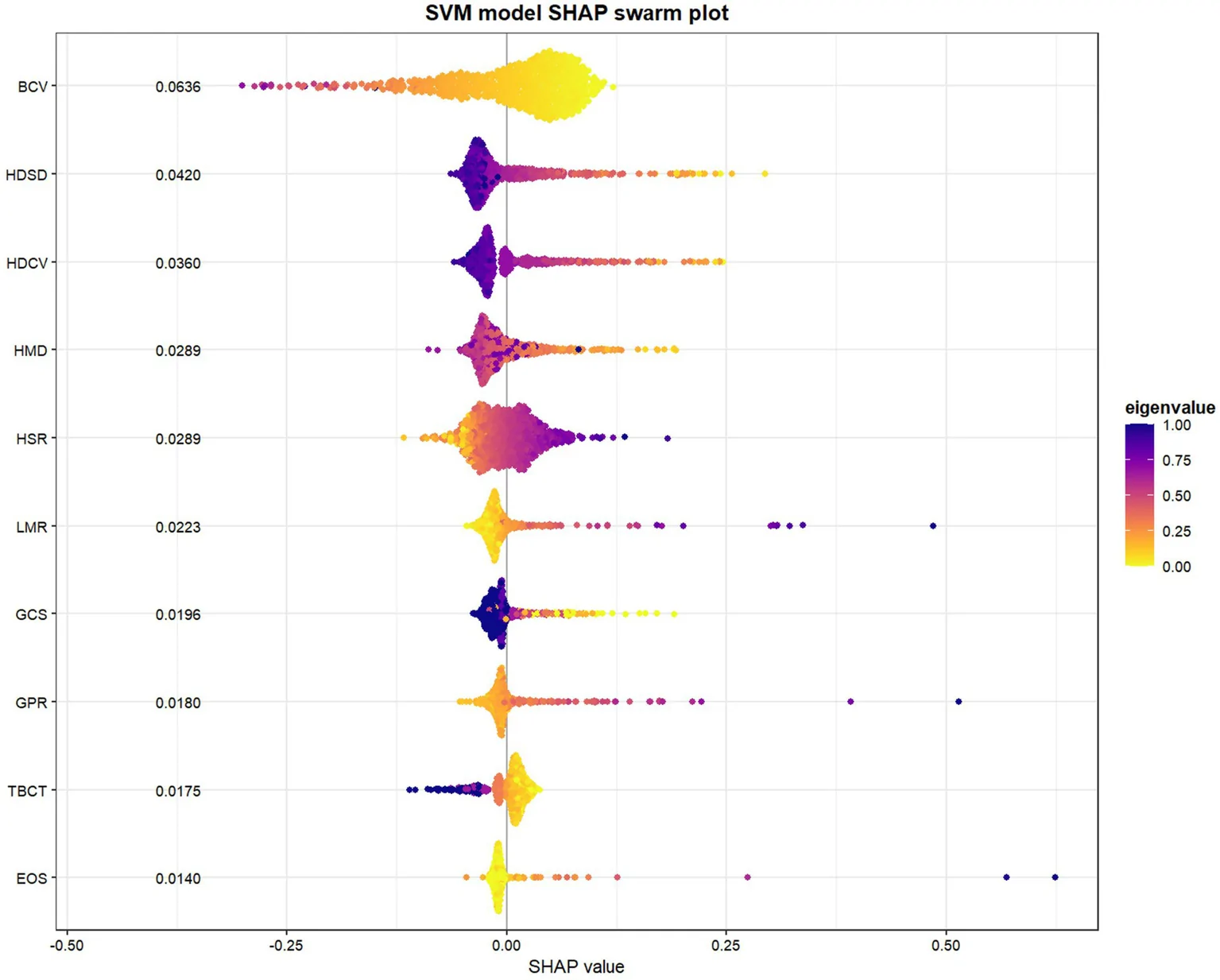
The SHAP beeswarm plot of the optimal SVM model.

To better determine the importance of each feature in the predictive model for DHP in frontal lobe contusion a SHAP beeswarm plot for the SVM model was constructed in this study (Figure 8). Feature importance ranking (Y-axis) indicates the significance of risk factors to the predictive model, while the SHAP value (X-axis) is an index reflecting the impact degree of a specific feature in the model. When the SHAp value is greater than 0, the corresponding feature value acts as a risk factor for the outcome, and a higher SHAP value indicates an increased risk of DHP. Each point represents the contribution of a specific feature to the model prediction for an individual patient. The color of the points varies according to the feature values: purple indicates higher feature values, while yellow indicates lower ones. Baseline contusion volume (BCV) had the highest mean absolute SHAP value (0.064), indicating that it possesses the strongest predictive power among all features and is the core predictive factor for DHP. Followed by imaging features including the standard deviation of hematoma density (HDSD), hematoma density coefficient of variation (HDCV), mean hematoma density (HMD), and baseline hematoma surface area-to-volume ratio (HSR). Among clinical and laboratory indicators, the lymphocyte-to-monocyte ratio (LMR), admission Glasgow Coma Scale (GCS) score, glucose-to-blood potassium ratio (GPR), time from onset to baseline CT (TBCT), and eosinophil count (EOS) are also important risk factors for DHP in frontal lobe contusion.

From a clinical perspective, the imaging predictors identified by SHAP are biologically plausible. Baseline contusion volume may reflect the initial burden of traumatic vascular injury, whereas HDSD, HDCV, and HMD may capture hematoma density heterogeneity related to mixed clot age, ongoing bleeding, or unstable coagulation. HSR may reflect irregular lesion morphology and a larger interface between injured tissue and surrounding brain. Among clinical and laboratory predictors, lower GCS score reflects greater neurological injury severity, shorter TBCT may indicate that the baseline scan was obtained during an early dynamic phase of hematoma evolution, GPR may reflect stress-related hyperglycemia and electrolyte disturbance, and LMR and EOS may reflect post-traumatic immune-inflammatory responses. These interpretations are clinically plausible but should be considered exploratory.

#### 3.3.2 SHAP dependence plots

**Figure 9:**
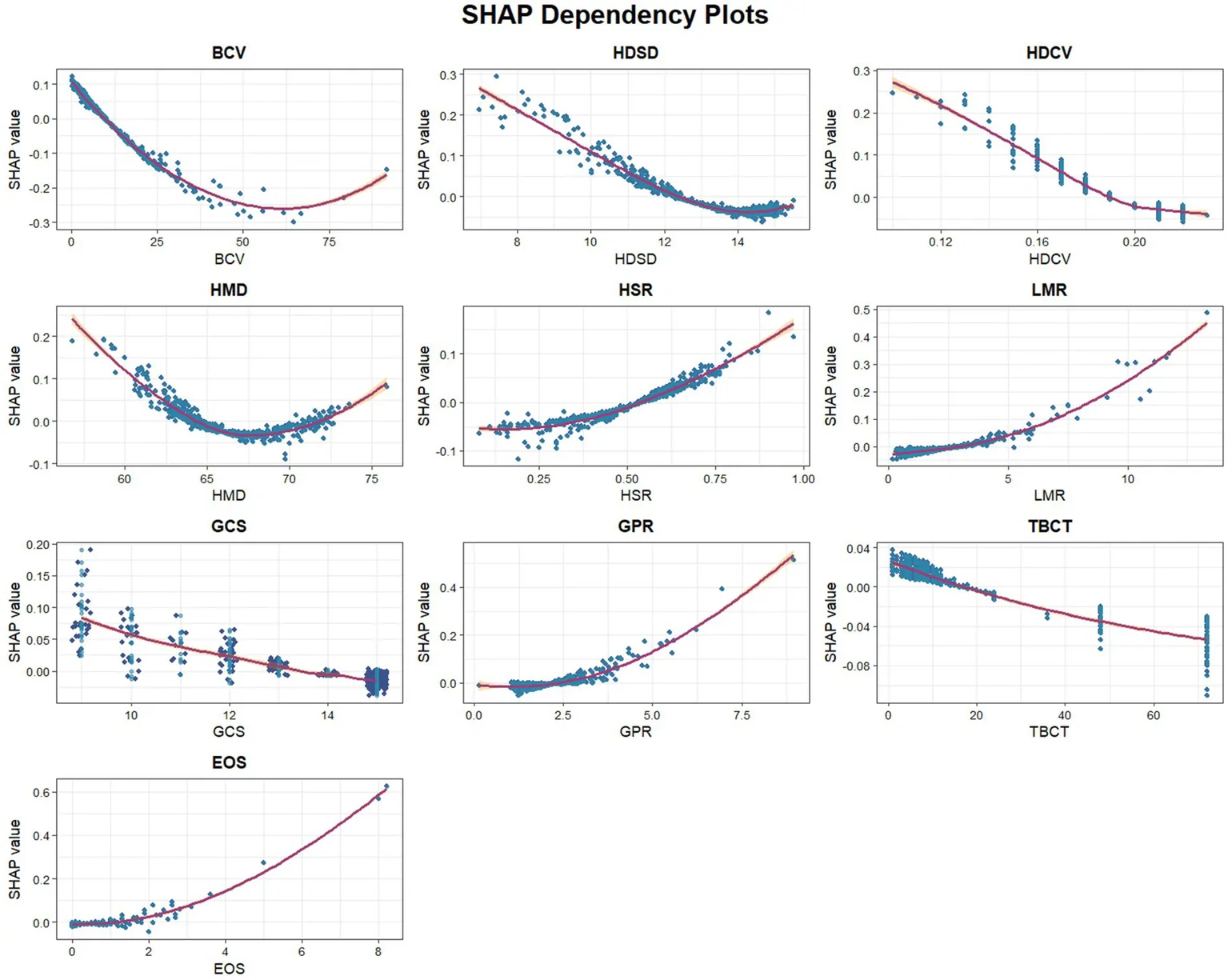
Presents the SHAP dependence plots of the SVM model.

Building on the overall feature importance identified by the SHAP beeswarm plot, SHAP dependence plots (Figure 9) further reveal the complex nonlinear relationships between individual core indicators and the risk of DHP. These plots integrate SHAP values with the values of each individual feature: when the SHAP value of a feature exceeds 0, it indicates an increased risk of DHP; conversely, a SHAP value less than 0 suggests a decreased risk. More specifically, the SHAP dependence plots suggested several exploratory model-derived inflection points associated with increased predicted risk of DHP, including BCV < 10 mL, HDSD <12, HDCV <0.19, HMD < 64 or >72, HSR > 0.5, LMR > 2.5, GCS score <14, GPR > 2.5, TBCT <18 h, and EOS > 0.2 × 10^9^/L. These values should be interpreted as hypothesis-generating model-derived patterns rather than biologically or clinically validated thresholds.

#### 3.3.3 Personalized prediction

**Figure 10:**
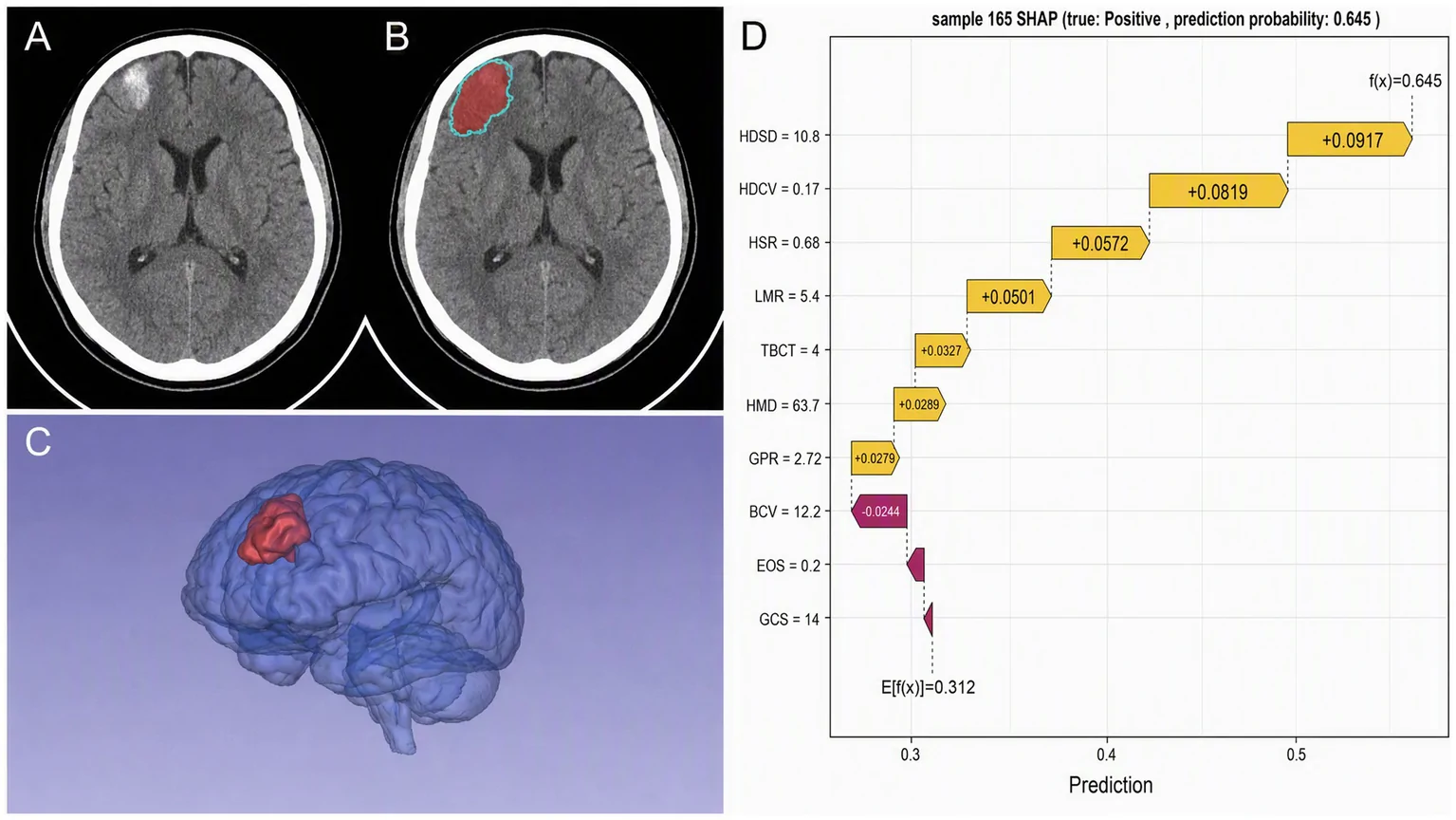
Individual prediction and SHAP interpretation for Patient 165, a patient with frontal lobe contusion and DHP. **(A)** Baseline CT image. **(B)** Segmentation of the hematoma and surrounding edema. **(C)** Three-dimensional reconstruction of the hematoma and surrounding edema. **(D)** SHAP force plot showing the contributions of individual features to the prediction.

The predictive model constructed by combining the SVM model with SHAP interpretable analysis enables the prediction of DHP risk in individual patients with frontal lobe contusion. In the single-sample SHAP contribution plots, yellow areas indicate that feature values increase the probability of DHP in frontal lobe contusion while red areas indicate that feature values decrease this probability. Specifically, f(x) represents the comprehensive SHAP value for each patient, and the baseline value denotes the average SHAP value across all samples. If the f(x) value exceeds the baseline value, the model predicts a higher likelihood of DHP in the patient. Interpretation of the single-sample SHAP contribution plots (Figure 10) revealed the following: For Patient 165 (a DHP-positive case with a predicted probability of 0.645), the primary positive contributing features were the standard deviation of hematoma density (HDSD, value: 10.8, SHAP contribution: +0.092), hematoma density coefficient of variation (HDCV, value: 0.17, SHAP contribution: +0.082), and lymphocyte-to-monocyte ratio (LMR, value: 5.4, SHAP contribution: +0.05). For comparison, Patient 471, a DHP-negative case not shown in Figure 10, had a predicted probability of 0.200, the main negative contributing features included time from onset to baseline CT (TBCT, value: 72 h, SHAP contribution: −0.053), mean hematoma density (HMD, value: 66.5, SHAP contribution: −0.028), and LMR (value: 0.83, SHAP contribution: −0.015). Thus, the SVM model may assist risk stratification to distinguish between DHP and non-progression cases, and provides individualized risk estimates based on individual patient characteristics. Additional examples of individual SHAP interpretation plots for more patients are provided in the Supplementary Figure S4.

### 3.4 Calibration and clinical utility

The calibration performance of the final SVM model was quantitatively assessed. In the internal test set, the calibration slope was 1.27, the calibration intercept was −0.074, the Brier score was 0.168, and the Hosmer–Lemeshow test *p* value was 0.025. In the external validation cohort, the calibration slope was 0.949, the calibration intercept was 0.672, the Brier score was 0.238, and the Hosmer–Lemeshow test *p* value was <0.001. Detailed calibration results are provided in Supplementary Table S14.

Decision-curve analysis showed that the SVM model provided a greater net benefit than the treat-all and treat-none strategies across a threshold probability range of approximately 0.1 to 0.7. This finding suggests potential clinical utility for risk stratification within this range; however, DCA does not prove improved clinical outcomes, and prospective clinical impact studies are required before implementation.

### 3.5 Sensitivity analyses

To evaluate the impact of patients with intermediate hematoma expansion (10–25% volume increment) on model predictive performance, we conducted a sensitivity analysis by incorporating an additional 30 patients with 10–25% hematoma growth into the original test set while preserving the primary cohort’s 1:9 case-to-control ratio. Two distinct labeling strategies were applied to these intermediate patients: Test0 categorized the 30 subjects as non-DHP, whereas Test1 reclassified the same cohort as DHP. For both outcome definitions, any hematoma volume increase exceeding 10% was defined as DHP, and growth ≤10% was defined as non-DHP.

The SVM model yielded an AUROC of 0.777 (95% CI: 0.717–0.837) for Test0 and 0.762 (95% CI: 0.705–0.820) for Test1, with comparable accuracy, sensitivity, specificity, precision, and F1-score observed across the two testing scenarios. These results were generally consistent with those from the primary analysis, suggesting that the predictive capacity of our SVM model remains robust with minimal performance fluctuation even when patients with mild-to-moderate hematoma expansion (10–25% volume increase) are included in the test dataset. Full quantitative performance metrics from the sensitivity analysis are summarized in Supplementary Figure S5.

To cross-validate the robustness of our dimensionality-reduction strategy, we constructed an auxiliary sensitivity model adopting a lenient inclusion cutoff: all variables selected by at least one screening algorithm (consensus frequency ≥1) were fed into the SVM pipeline for benchmarking. The discriminatory performance of this expanded high-dimensional feature set was quantified and contrasted against the streamlined 10-predictor baseline model. A marginal AUROC discrepancy across the two model specifications would empirically confirm that aggressive feature pruning incurs negligible predictive degradation, yet yields tangible improvements in model simplicity, lower overfitting risk, and superior real-world clinical operability.

## 4 Discussion

The occult progression of frontal lobe contusion is widely recognized as a notorious clinical challenge in neurosurgery. Patients with this condition are typically characterized by mild neurological abnormalities in the early post-injury phase; yet, pathological processes triggered by contusional lesions—such as microvascular tearing and inflammatory activation—may lead to acute hematoma expansion within a short period. This rapid expansion can subsequently induce life-threatening complications, including acute intracranial hypertension and cerebral herniation. Currently, there is a lack of validated predictive tools specifically for DHP in frontal lobe contusion and traditional assessment methods fail to meet the clinical needs for precise diagnosis and treatment.

Recently, leveraging their superior capabilities in information analysis, comprehensive assessment strategies, and enhanced predictive efficacy, machine learning approaches have been increasingly adopted to explore the feasibility and applicability of prognostic prediction in TBI. Abujaber et al. (2020) confirmed using national trauma registry data that the support vector machine (SVM) model significantly outperformed traditional multivariable analysis models in predicting in-hospital mortality among patients with traumatic brain injury. Bark et al. (2024) validated through an international multicenter cohort that random forest (RF) exhibits robust cross-population adaptability in TBI prognostic prediction. Duan et al. (2022), after comparing seven machine learning algorithms, identified that SVM and k-nearest neighbor (KNN) demonstrated the optimal performance in predicting intracerebral hemorrhage expansion. Building on this evidence, the present study systematically incorporated 10 robust and representative algorithms in the current medical prediction field—including XGBoost, random forest (RF), support vector machine (SVM), and boosted regression tree (BRT)—to develop a predictive model for DHP in frontal lobe contusion.

Notably, the robustness of a predictive model hinges on rigorous methodological procedures, particularly in the selection of predictive variables. In this study, we constructed a multidimensional indicator system for variable selection, taking the pathophysiological mechanisms of frontal lobe contusion as the starting point and integrating the feature selection results in Figure 4 and the correlation heatmap in Supplementary Figure S3. From the perspective of pathophysiological mechanisms: First, frontal lobe contusion inevitably triggers coagulopathy-fibrinolysis imbalance, which is the key pathological basis for sustained hematoma expansion. Therefore, this study included core coagulation indicators such as fibrinogen, D-dimer, and prothrombin time.

Meanwhile, in line with clinical research hotspots, we calculated derivative indicators including the D-dimer-to-fibrinogen ratio (DFR) focused on by Wang et al. (2024), to more accurately reflect coagulation function status (Shafiei et al., 2023). Second, regarding the inflammatory response, the frontal lobe is enriched with immunocompetent cells; post-traumatic central immune activation in this region is more pronounced, which is prone to inducing immune dysregulation and exacerbating blood–brain barrier disruption. Based on this characteristic, we included a series of inflammation-related indicators such as white blood cells and neutrophils, and constructed combined indicators—including the lymphocyte-to-monocyte ratio (LMR) and neutrophil-to-lymphocyte ratio (NLR)—which have been confirmed by Reznik et al. (2021) to be associated with adverse prognosis in critical cerebrovascular diseases, so as to comprehensively assess the post-traumatic immune-inflammatory imbalance status (Zhu, 2022). Third, metabolic disorders are common complications after brain injury and are closely related to patient prognosis. Thus, this study also included indicators such as glucose and blood potassium, and cited the glucose-to-blood potassium ratio (GPR), a derivative indicator reported by Shibata et al. (2021), to comprehensively reflect the patient’s metabolic status. Additionally, Ji et al. (2025) identified that baseline hematoma volume and mean hematoma density are associated with DHP. Innovatively, we introduced a set of imaging indicators as predictive variables, including baseline hematoma volume, baseline hematoma surface area, baseline hematoma surface area-to-volume ratio (HSR), mean hematoma density (HMD), standard deviation of hematoma density (HDSD), and hematoma density coefficient of variation (HDCV). Ultimately, a total of 74 clinically accessible variables were collected in this study, which can comprehensively capture the complex early-stage characteristics of frontal lobe contusion.

To avoid model overfitting caused by variable redundancy, this study adopted a multidimensional feature selection strategy referenced from Song et al. (2021). Specifically, seven complementary methods—including maximum relevance minimum redundancy (mRMR), extreme gradient boosting (XGBoost), and recursive feature elimination (RFE)—were comprehensively utilized. Combined with risk factors reported in previous literature and clinical expert judgments, 10 core predictive factors were ultimately screened out from the 74 candidate variables. The dataset was randomly split into a training set and a test set at a 7:3 ratio, and hyperparameter tuning was performed using grid search combined with five-fold cross-validation. Consistently with the “multi-method screening-cross-validation” workflow adopted by Xia et al. (2022) in their hematoma expansion prediction model, this protocol can effectively reduce the risk of overfitting and enhance model stability. In the model evaluation and validation phase, multiple dimensional metrics were employed for comprehensive assessment, including area under the receiver operating characteristic curve (AUC), accuracy, sensitivity, specificity, and F1-score, to fully reflect the predictive performance of the models. Meanwhile, to address the “black box” problem inherent in traditional machine learning models, the SHAP framework was utilized for interpretability analysis in this study. Visualization plots such as SHAP beeswarm plots, dependence plots, and single-sample contribution plots were generated to clearly elucidate the importance of each feature and their intrinsic associations with DHP. This approach renders the model’s predictive logic more comprehensible and acceptable to clinicians (Shakya et al., 2025), which is highly consistent with the approach adopted by Wei et al. (2025) who applied SHAP to enhance interpretability in their predictive model for spontaneous intracerebral hemorrhage.

In-depth interpretation of the optimal SVM model not only validated certain classical theories but also, more importantly, revealed novel patterns underlying DHP in frontal lobe contusion. In the predictive model developed in this study, imaging features were confirmed as the most crucial factors for predicting DHP. SHAP analysis identified baseline contusion volume (BCV) as the most influential predictive factor. Distinct from the conclusions of previous studies (Åkerlund and Holst, 2024; Chang et al., 2006), SHAP dependence analysis clearly demonstrated a significant nonlinear relationship between baseline contusion volume and progression risk—specifically, BCV < 10 mL was associated with an increased risk of DHP. This finding is consistent with the results reported by Cepeda et al. (2015) and supports the observation by Jirlow et al., 2024 that “the risk decreases when hematoma volume exceeds 15 mL.” Small hematomas possess greater expansion potential within the limited anatomical space of the frontal lobe, and initial small hematomas may represent injury foci in the active bleeding phase. Consequently, this finding suggests that neurosurgeons should maintain a high level of vigilance for small-volume frontal lobe contusions on initial cranial CT scans. Hematoma density heterogeneity indicators—standard deviation of hematoma density (HDSD) and hematoma density coefficient of variation (HDCV)—also exhibited substantial predictive value. SHAP analysis suggested a model-derived inflection point of approximately 12 HU for HDSD, with values below this level associated with increased predicted risk in the fitted model. This pattern should be interpreted as exploratory rather than as a clinically validated threshold (Oge et al., 2020). A risk inflection point for HDCV was identified around 0.19, which reflects the balance between coagulation and fibrinolysis processes within the hematoma. Correlation analysis further revealed a strong negative correlation between BCV and all density indicators, leading to the definition of a frontal lobe-specific high-risk hematoma pattern: “small volume with high heterogeneity.” This pattern is a typical imaging manifestation of “delayed hematoma” in the frontal lobe, which is highly consistent with the theory proposed by Ji et al. (2025) that “quantitative shape irregularity and density heterogeneity predict hematoma expansion.” The present study validated the applicability of this theory in frontal lobe contusion using machine learning approaches. Additionally, the inclusion of baseline hematoma surface area-to-volume ratio (HSR) further corroborated from a morphological perspective that irregular, more permeable hematomas are associated with a higher progression risk. This finding echoes the conclusion by Zhang S. Q. et al. (2024) that hematoma surface area is closely correlated with the growth of perilesional edema.

Beyond imaging features, the evaluation of systemic response indicators in this study yielded findings of significant theoretical value. Regarding the inflammatory response, in sharp contrast to Zhang H. et al. (2024) who, based on monocyte-to-lymphocyte ratio (MLR), concluded that “elevated MLR levels are independently associated with the risk of DHP” in their study on acute traumatic intracerebral hemorrhage expansion after cerebral contusion, the feature importance ranking in this study clearly identified that the previously described lymphocyte-to-monocyte ratio (LMR) has superior predictive efficacy. This discrepancy could stem from the unique immunology of the frontal lobe, an area rich in resident microglia. Their rapid post-traumatic activation may create a distinct immune microenvironment. In this context, a decreased LMR might more accurately reflect lymphocyte exhaustion and a state of immunosuppression, potentially a key driver of DHP specific to the frontal lobe, as opposed to a general inflammatory response indicated by MLR. Meanwhile, the independent predictive value of eosinophils (EOS) supports the emerging perspective that eosinophils act as active effector cells in acute brain injury. They directly impair blood–brain barrier integrity by releasing cytotoxic substances such as eosinophil cationic protein (Wang et al., 2025; Zhang D. et al., 2024), which aligns with the value of leukocyte clustering in TBI prognosis assessment reported by Wang et al. (2025). In terms of metabolic indicators, this study redefined the clinical value of the glucose-to-blood potassium ratio (GPR). Unlike Tsuchiya et al. (2023) who utilized GPR as a predictive indicator for long-term mortality in critically ill TBI patients, the present study found that GPR exhibits early warning value for DHP even at a lower threshold (> 2.5). This indicates that the stress-induced metabolic disorders represented by GPR are initiated in the very early stage of the disease and directly participate in the pathological process of DHP. Elevated GPR not only reflects catecholamine-driven stress hyperglycemia but also implies potassium disorders caused by sodium-potassium pump dysfunction in cell membranes. Such changes in GPR may be primarily induced by the combined effects of overactivation of the hypothalamic–pituitary–adrenal axis and autonomic nervous system dysfunction. Second, a shorter time from onset to baseline CT (TBCT < 18 h) was associated with a higher progression risk, which confirms the clinical characteristic that frontal lobe hematomas mostly occur in the early post-injury period and evolve continuously (Shafiei et al., 2023). This is consistent with the conclusion reported by Moyer et al. (2022) that “the first 24 h after traumatic brain injury represents a critical time window for emergency neurosurgical intervention.” Furthermore, a lower admission Glasgow Coma Scale (GCS) score (< 14 points), as an objective indicator of neurological function injury, showed predictive value highly consistent with clinical cognition. A higher GCS score indicates milder consciousness disturbance, smaller areas of brain tissue and vascular injury, and a weaker pathological basis for DHP (Wang et al., 2024).

Having reviewed the predictive value of key individual indicators, our comprehensive comparison of all 10 machine learning models provides the overarching conclusion: SVM provided the highest point estimate and optimal sensitivity, though without statistical superiority over all alternatives. Nevertheless, its AUROC confidence interval overlapped with those of several competing models, and DeLong testing did not confirm statistically significant superiority over all alternatives. Therefore, the selection of SVM should be interpreted as a pragmatic choice based on overall performance, clinical screening needs, and interpretability rather than definitive statistical superiority. The superiority of the SVM model can be explained by several factors pertinent to both the clinical question and the underlying data characteristics. First, its high predictive efficacy, particularly the sensitivity of 89.9%, directly addresses the paramount clinical need in managing frontal contusions—avoiding missed diagnoses in a condition with occult presentation. This performance (AUC = 0.801, 95% CI: 0.742–0.859) significantly surpassed that of other models in our test (e.g., KNN AUC = 0.658, DT AUC = 0.650) and outperforms previous models generalized to all contusions (often AUC < 0.75; Lee et al., 2024; Li et al., 2022). Specifically, Sheng et al. (2022) clinical predictive nomogram for traumatic brain parenchyma hematoma progression was not optimized for the frontal lobe, while Li et al. (2022) prognostic model for cerebral contusion relied on a general cohort, both yielding AUC values below 0.75. In contrast, our SVM model to a potentially useful level for auxiliary risk stratification, with a sensitivity further improved compared to Lee et al. (2024) XGBoost model for TBI hemorrhage progression. The clinical principle of “prioritizing sensitivity over specificity” in screening high-risk patients is thus well-served, even with a specificity of 0.571, as it aligns with the core clinical goal of minimizing missed diagnoses in this insidious condition (Moyer et al., 2022). Second, the data adaptability of SVM through nonlinear kernel functions (e.g., RBF) is particularly suited to the complex, multifactorial pathophysiology of DHP. This condition likely involves synergistic or antagonistic interactions among predictors, which linear models (e.g., logistic regression) or simpler nonlinear models prone to overfitting (e.g., decision trees) may fail to capture effectively. As supported by Duan et al. (2022), SVM’s ability to map data into higher-dimensional space allows it to model these intricate relationships more robustly—an advantage over KNN and DT, both of which struggle to adapt to the heterogeneous data landscape of frontal lobe contusion. Third, the clinical utility of the model is enhanced by the practicality of its input features. All 10 core predictors are derived from routine admission assessments, requiring no specialized or costly additional tests. This stands in contrast to radiomics-based models that depend on complex feature extraction (Shih et al., 2022) and aligns with the practical application orientation emphasized by Abujaber et al. (2020) for TBI prediction tools. Because all predictors are routinely available, the model may be feasible for further evaluation in different healthcare settings. However, its applicability outside Chinese tertiary hospitals remains uncertain and requires additional validation. Finally, the integration with the SHAP framework resolves the traditional “black box” limitation of machine learning, achieving a balance between high accuracy and interpretability (Wei et al., 2025), an approach validated by Wei et al. (2025) in their predictive model for spontaneous intracerebral hemorrhage. The SHAP plots provide actionable insights: the beeswarm plot quantifies and ranks feature importance (e.g., baseline volume as the top predictor with a mean absolute SHAP value of 0.064), the dependence plots reveal potentially useful level for auxiliary risk stratification (e.g., HDSD < 12 HU), and the force plots enable individualized risk decomposition. This transparency not only aids clinical decision-making but also facilitates communication with patients and families (Liu et al., 2024), while overcoming the limitations of traditional linear statistical models that cannot capture such nonlinear relationships or provide granular interpretive insights (Wang et al., 2022).

Furthermore, the model did not incorporate potential influential factors such as genetic polymorphisms, dynamic imaging data, or detailed therapeutic interventions, which might affect predictive accuracy. Although SHAP analysis enhanced interpretability, the biological mechanisms underlying certain revealed nonlinear relationships still require experimental validation. Although an external validation cohort was included, both cohorts were obtained from Chinese tertiary hospitals. Therefore, the generalizability of the model to other geographic regions, ethnic populations, hospital levels, and healthcare systems remains uncertain. To address these limitations, future studies should validate and update the model using prospective, multicenter, and internationally diverse cohorts. Expanding the data dimension by integrating genetic markers, serum biomarkers, and dynamic imaging data could enhance the comprehensiveness of predictive factors. Developing adaptive models using reinforcement learning techniques and conducting intervention trials to validate the effectiveness of risk-stratified management are also warranted. Ultimately, these efforts aim to provide a high-level evidence-based foundation for the precise treatment of frontal lobe contusion.

## 5 Conclusion

This study developed and externally validated an interpretable multimodal machine learning model for early prediction of DHP in patients with frontal lobe contusion. Ten readily available clinical, laboratory, and imaging indicators were identified as key predictors. The SVM model demonstrated moderate external performance and may assist early risk stratification and clinical monitoring. However, further prospective, multicenter, and geographically diverse validation is required before broad clinical implementation.

## Data Availability

The raw data supporting the conclusions of this article will be made available by the authors, without undue reservation.
