# Supplementary material for: An interpretable multimodal model for early prediction of delayed hematoma progression in frontal lobe contusion: a machine learning approach
Source: Front Neurosci. 2026 Aug 12;20:1897735. doi: 10.3389/fnins.2026.1897735 (PMC13506746; doi:10.3389/fnins.2026.1897735)
Supplement: Supplementary file 1 [file Supplementary_file_1.docx]

Supplementary materials

**Supplementary material**

**Table S1** Variable assignment and description**.**

**Table S2** Top 10 variables selected by 7 methods

**Table S3** Ranking of All Variable Importances

**Table S4** Key parameters of the Support Vector Machine (SVM) model

**Table S5** Key parameters of the K Nearest Neighbors (KNN) model

**Table S6** Key parameters of the Decision Tree model

**Table S7** Key parameters of the Artificial Neural Networks (ANNs) model

**Table S8** Key parameters of the Naïve Bayes model

**Table S9** Key parameters of the Random Forest model

**Table S10** Key parameters of the Boosted Regression Tree (BRT) model

**Table S11** Key parameters of the Light Gradient Boosting Machine (LightGBM) model

**Table S12** Key parameters of the Logistic Regression model

**Table S13** Key parameters of the eXtreme Gradient Boosting (XGBoost) model

**Table S14** Full performance metrics of all candidate machine learning models in internal test set and external validation cohort

**Table S15** The DeLong test compares results across the models

**Table S16 Completed TRIPOD+AI checklist for the present study**

**Fig.S1** Variable Selection Importance Ranking of BRT

**Fig.S2** Path Coefficient Diagram pf [Ridge Regression](https://blog.csdn.net/ChaneMo/article/details/142390915)

**Fig.S4** Shapley additive explanation plots, applicable to patients with frontal lobe contusion hematoma progression and non-progression.

**Fig.S5. ROC curves of the SVM model in sensitivity analysis incorporating patients with intermediate hematoma expansion (10%–25% volume increase)**

**Fig.S6. ROC curve of the SVM model constructed using features with consensus selection frequency ≥ 1**

**Table S1** Variable assignment and description

| **variable_name** | **original_description** | **new_clear_description** |
| --- | --- | --- |
| Hematoma expansion (Group) | No =0, Yes=1 | Binary outcome variable indicating whether the patient experienced hematoma expansion (0 = no expansion, 1 = expansion; defined as ≥25% increase in hematoma volume on follow-up CT scan within 72 hours after injury) |
| Age | Age | Continuous variable representing the patient's age in years at the time of hospital admission |
| Gender | Female=0, Male=1 | Binary variable for patient gender (0 = female, 1 = male) |
| contusion.Site | Single=0, Double=1 | Binary variable indicating the number of brain contusion sites (0 = single contusion site, 1 = multiple/double contusion sites) |
| SAH(Subarachnoid hemorrhage) | No=0, Yes=1 | Binary variable indicating the presence of subarachnoid hemorrhage (0 = no, 1 = yes; bleeding into the subarachnoid space of the brain) |
| SDH(Subdural Hemorrhage) | No=0, Yes=1 | Binary variable indicating the presence of subdural hemorrhage (0 = no, 1 = yes; bleeding between the brain's dura mater and arachnoid mater layers) |
| EDH(Extradural Hematoma) | No=0, Yes=1 | Binary variable indicating the presence of extradural (epidural) hematoma (0 = no, 1 = yes; bleeding between the skull and the dura mater layer of the brain) |
| Skull.Fracture | No=0, Yes=1 | Binary variable indicating the presence of a skull fracture (0 = no skull fracture, 1 = skull fracture confirmed on CT imaging) |
| Time.to.baseline.CT | Time.to.baseline.CT | Continuous variable representing the time interval (in hours) from brain injury to the acquisition of the baseline (admission) CT scan |
| Midline.shift | Midline.shift | Binary variable indicating the presence of midline shift of the brain (0 = no midline shift, 1 = midline shift; displacement of the brain's central structures due to mass effect from hematoma or edema) |
| Intraventricular.compression | No=0, Yes=1 | Binary variable indicating the presence of intraventricular compression (0 = no compression, 1 = compression; compression of the brain's ventricles caused by adjacent hematoma or edema) |
| Widening.and.deepening.of.cerebral.sulcus | No=0, Yes=1 | Binary variable indicating the presence of widening and deepening of cerebral sulci (0 = no, 1 = yes; a CT sign of brain atrophy or edema-related changes in the brain's surface grooves) |
| Shallow.fissure.fission.in.the.cerebral.sulcus | No=0, Yes=1 | Binary variable indicating the presence of shallow fissure fission in cerebral sulci (0 = no, 1 = yes; a CT imaging sign of altered morphology in the brain's surface grooves) |
| Black.hole.sign | No=0, Yes=1 | Binary variable indicating the presence of the black hole sign on CT imaging (0 = no, 1 = yes; a hypodense (dark) area within the hematoma, indicating active bleeding or coagulopathy, a well-known predictor of hematoma expansion) |
| Island.sign | No=0, Yes=1 | Binary variable indicating the presence of the island sign on CT imaging (0 = no, 1 = yes; small, separate hyperdense (bright) hematoma fragments within a larger hypodense area, a sign of active bleeding and high risk of hematoma expansion) |
| Blend.sign | No=0, Yes=1 | Binary variable indicating the presence of the blend sign on CT imaging (0 = no, 1 = yes; a mix of hypodense and hyperdense areas within the hematoma, indicating a combination of acute and subacute bleeding, a predictor of hematoma expansion) |
| Satellite.sign | No=0, Yes=1 | Binary variable indicating the presence of the satellite sign on CT imaging (0 = no, 1 = yes; small, separate hematoma lesions adjacent to the main contusion site, indicating multiple bleeding foci) |
| Baseline.Crebral.edema.volume | Baseline.Crebral.edema.volume | Continuous variable representing the volume (in cubic centimeters, cm³) of cerebral edema measured on the baseline admission CT scan (note: "Crebral" is a typo for "Cerebral") |
| Baseline.Contusion.volume | Baseline.Contusion.volume | Continuous variable representing the volume (in cubic centimeters, cm³) of the brain contusion/hematoma measured on the baseline admission CT scan |
| HMD | Hematoma.mean.density | Continuous variable representing the mean density (in Hounsfield Units, HU) of the hematoma on baseline CT imaging, reflecting the consistency and age of the blood clot |
| HDSD | Hematoma.density.SD | Continuous variable representing the standard deviation (SD) of hematoma density (in Hounsfield Units, HU) on baseline CT imaging, reflecting heterogeneity within the hematoma (a marker of active bleeding risk) |
| Baseline.Contusion.Surface.area | Baseline.Contusion.Surface.area | Continuous variable representing the surface area (in square centimeters, cm²) of the brain contusion/hematoma measured on the baseline admission CT scan |
| HSR | Hematoma.SR | Continuous variable representing the hematoma shape regularity (SR) index, a measurement of how regular or irregular the shape of the hematoma is on CT imaging (lower values = more irregular shape, higher expansion risk) |
| HDCV | Hematoma.DCV | Continuous variable representing the hematoma density coefficient of variation (DCV), calculated as (hematoma density SD / mean hematoma density), a normalized measure of hematoma heterogeneity |
| Hypertension | No=0, Yes=1 | Binary variable indicating a history of hypertension (0 = no history of hypertension, 1 = diagnosed hypertension per medical records) |
| Coronary.heart.disease | No=0, Yes=1 | Binary variable indicating a history of coronary heart disease (0 = no history, 1 = diagnosed coronary heart disease per medical records) |
| Diabetes | No=0, Yes=1 | Binary variable indicating a history of diabetes mellitus (0 = no history, 1 = diagnosed diabetes per medical records) |
| GCS | Glasgow Coma Scale | Continuous variable representing the Glasgow Coma Scale (GCS) score on hospital admission, a 3-15 point scale measuring the severity of traumatic brain injury (lower scores = more severe neurological impairment) |
| Temperature | Temperature | Continuous variable representing the patient's body temperature (in degrees Celsius, °C) on hospital admission |
| Heart.rate | Heart.rate | Continuous variable representing the patient's heart rate (in beats per minute, bpm) on hospital admission |
| Systolic.blood.pressure | Systolic.blood.pressure | Continuous variable representing the patient's systolic blood pressure (in millimeters of mercury, mmHg) on hospital admission |
| Diastolic.blood.pressure | Diastolic.blood.pressure | Continuous variable representing the patient's diastolic blood pressure (in millimeters of mercury, mmHg) on hospital admission |
| White.blood.cell | White.blood.cell | Continuous variable representing the patient's white blood cell (WBC) count (in 10⁹ cells per liter, 10⁹/L) from the admission blood test, a marker of systemic inflammation |
| Neutrophil | Neutrophil | Continuous variable representing the patient's neutrophil count (in 10⁹ cells per liter, 10⁹/L) from the admission blood test, the most common type of white blood cell and a key marker of bacterial infection or inflammation |
| Lymphocyte | Lymphocyte | Continuous variable representing the patient's lymphocyte count (in 10⁹ cells per liter, 10⁹/L) from the admission blood test, a type of white blood cell involved in immune response |
| Monocyte | Monocyte | Continuous variable representing the patient's monocyte count (in 10⁹ cells per liter, 10⁹/L) from the admission blood test, a type of white blood cell involved in inflammation and immune regulation |
| Eosinophil | Eosinophil | Continuous variable representing the patient's eosinophil count (in 10⁹ cells per liter, 10⁹/L) from the admission blood test, a type of white blood cell involved in allergic reactions and parasitic infections |
| Basophil | Basophil | Continuous variable representing the patient's basophil count (in 10⁹ cells per liter, 10⁹/L) from the admission blood test, the rarest type of white blood cell involved in allergic reactions |
| Red.blood.cell | Red.blood.cell | Continuous variable representing the patient's red blood cell (RBC) count (in 10¹² cells per liter, 10¹²/L) from the admission blood test, responsible for oxygen transport in the blood |
| Hemoglobin | Hemoglobin | Continuous variable representing the patient's hemoglobin level (in grams per liter, g/L) from the admission blood test, the protein in red blood cells that carries oxygen |
| RDW | red blood cell distribution width | Continuous variable representing the red blood cell distribution width (RDW), a measure of variation in the size of red blood cells from the admission blood test |
| Platelet | Platelet | Continuous variable representing the patient's platelet count (in 10⁹ cells per liter, 10⁹/L) from the admission blood test, blood cells involved in clotting and hemostasis |
| Blood.Potassium | Blood.Potassium | Continuous variable representing the patient's blood potassium level (in millimoles per liter, mmol/L) from the admission blood test, an electrolyte critical for heart and muscle function |
| Blood.sodium | Blood.sodium | Continuous variable representing the patient's blood sodium level (in millimoles per liter, mmol/L) from the admission blood test, the main electrolyte regulating fluid balance in the body |
| urea | Urea | Continuous variable representing the patient's blood urea nitrogen (BUN) level (in millimoles per liter, mmol/L) from the admission blood test, a waste product filtered by the kidneys, a marker of kidney function |
| Creatinine | Creatinine | Continuous variable representing the patient's blood creatinine level (in micromoles per liter, μmol/L) from the admission blood test, a waste product from muscle metabolism, a key marker of kidney function |
| Blood.glucose | Blood.glucose | Continuous variable representing the patient's blood glucose level (in millimoles per liter, mmol/L) from the admission blood test, the amount of sugar in the blood |
| uric.acid | uric.acid | Continuous variable representing the patient's blood uric acid level (in micromoles per liter, μmol/L) from the admission blood test, a waste product from purine metabolism |
| Total.bilirubin | Total.bilirubin | Continuous variable representing the patient's total bilirubin level (in micromoles per liter, μmol/L) from the admission blood test, a waste product from red blood cell breakdown, a marker of liver function |
| Total.protein | Total.protein | Continuous variable representing the patient's total blood protein level (in grams per liter, g/L) from the admission blood test, including albumin and globulin, a marker of nutritional and liver status |
| Albumin | Albumin | Continuous variable representing the patient's blood albumin level (in grams per liter, g/L) from the admission blood test, the main protein in blood plasma, a marker of nutritional status and liver function |
| AST | Aspartate aminotransferase | Continuous variable representing the patient's aspartate aminotransferase (AST) level (in units per liter, U/L) from the admission blood test, an enzyme found in the liver, heart, and muscles, a marker of tissue damage |
| ALT | Alanine aminotransferase | Continuous variable representing the patient's alanine aminotransferase (ALT) level (in units per liter, U/L) from the admission blood test, an enzyme found primarily in the liver, a key marker of liver damage |
| PT | Prothrombin Time | Continuous variable representing the patient's prothrombin time (PT) (in seconds) from the admission blood test, a measure of how long it takes for blood to clot, used to assess coagulation function |
| INR | International Normalized Ratio | Continuous variable representing the patient's International Normalized Ratio (INR), a standardized measure of prothrombin time used to monitor blood clotting status, especially in patients on anticoagulant medication |
| APTT | Activated partial thromboplastin time | Continuous variable representing the patient's activated partial thromboplastin time (APTT) (in seconds) from the admission blood test, a measure of blood clotting time used to assess the intrinsic coagulation pathway |
| FIB | Fibrinogen | Continuous variable representing the patient's fibrinogen level (in grams per liter, g/L) from the admission blood test, a protein in the blood that is converted to fibrin during clot formation, a key coagulation factor |
| TT | thrombin time | Continuous variable representing the patient's thrombin time (TT) (in seconds) from the admission blood test, a measure of the time it takes for fibrinogen to convert to fibrin, used to assess fibrinogen function and coagulation |
| D.dimer | D-Dimer | Continuous variable representing the patient's D-dimer level (in milligrams per liter, mg/L) from the admission blood test, a breakdown product of fibrin clots, a marker of active blood clot formation and breakdown |
| SII | Systemic Immune-Inflammation Index | Continuous variable representing the Systemic Immune-Inflammation Index (SII), calculated as (platelet count × neutrophil count) / lymphocyte count, a marker of systemic inflammation and immune status |
| NLR | neutrophil-to-lymphocyte ratio | Continuous variable representing the neutrophil-to-lymphocyte ratio (NLR), calculated as (neutrophil count / lymphocyte count), a widely used marker of systemic inflammation and stress response |
| SIRI | Systemic Inflammatory Response Index | Continuous variable representing the Systemic Inflammatory Response Index (SIRI), calculated as (neutrophil count × monocyte count) / lymphocyte count, a marker of systemic inflammatory response severity |
| PLR | Platelet-lymphocyte ratio | Continuous variable representing the platelet-to-lymphocyte ratio (PLR), calculated as (platelet count / lymphocyte count), a marker of inflammation and immune status |
| DFR | D-dimer to fibrinogen ratio | Continuous variable representing the D-dimer-to-fibrinogen ratio (DFR), calculated as (D-dimer level / fibrinogen level), a marker of coagulation activation and fibrinolytic activity |
| MLR | Monocyte to Lymphocyte Ratio | Continuous variable representing the monocyte-to-lymphocyte ratio (MLR), calculated as (monocyte count / lymphocyte count), a marker of inflammation and immune regulation |
| LMR | Lymphocyte to Monocyte Ratio | Continuous variable representing the lymphocyte-to-monocyte ratio (LMR), calculated as (lymphocyte count / monocyte count), a marker of immune cell balance and inflammation |
| RPR | Red cell distribution width-to-platelet ratio | Continuous variable representing the red cell distribution width-to-platelet ratio (RPR), calculated as (RDW value / platelet count), a marker of inflammation and nutritional status |
| PNI | Prognostic Nutritional Index | Continuous variable representing the Prognostic Nutritional Index (PNI), calculated as (10 × albumin level) + (0.005 × lymphocyte count), a marker of nutritional status and immune function |
| APRI | Aspartate Aminotransferase to Platelet Ratio Index | Continuous variable representing the Aspartate Aminotransferase to Platelet Ratio Index (APRI), calculated as (AST level / upper limit of normal AST) / platelet count, a marker of liver fibrosis and damage |
| GPR | Serum glucose potassium ratio | Continuous variable representing the serum glucose-potassium ratio (GPR), calculated as (blood glucose level / blood potassium level), a marker of metabolic stress and inflammation |
| uHG | Ultra-early Hematoma Growth | Continuous variable representing ultra-early hematoma growth (uHG), defined as the increase in hematoma volume (in cm³) within the first 6 hours after injury, a marker of active bleeding |
| Anterior.horns.of.the.bilateral.lateral.ventricles | Anterior.horns.of.the.bilateral.lateral.ventricles | Binary variable indicating the status of the anterior horns of the bilateral lateral ventricles (0 = normal morphology, 1 = compressed/abnormal morphology; a CT sign of increased intracranial pressure) |
| Mean.arterial.pressure | Mean.arterial.pressure | Continuous variable representing the patient's mean arterial pressure (MAP) (in millimeters of mercury, mmHg) on hospital admission, calculated as (2 × diastolic blood pressure + systolic blood pressure) / 3, a measure of average blood pressure during a cardiac cycle |
| Ultra.early.edema.expansion.rate | Ultra.early.edema.expansion.rate | Continuous variable representing the ultra-early edema expansion rate, calculated as the change in cerebral edema volume per hour within the first 24 hours after injury, a marker of edema progression speed |

**Fig. S1** Variable Selection Importance Ranking of BRT

This BRT-based variable selection chart shows the importance of various clinical and imaging variables in predicting 'frontal lobe contusion hematoma progression.

**Fig. S2** Visualization results of ridge regression. **A.** Regularization Path Plot. **B.**Cross-Validation Curve.

**Table S2** Top 10 variables selected by 7 methods

| lasso | Ridge Regression | Elastic-Net | MRMR | brt | xgb | rfe |
| --- | --- | --- | --- | --- | --- | --- |
| Hematoma.DCV | Hematoma.DCV | Baseline.Contusion.volume | Hematoma.DCV | Hematoma.mean.density | Hematoma.mean.density | Baseline.Crebral.edema.volume |
| Eosinophil | Hematoma.SR | Hematoma.DCV | LMR | Baseline.Crebral.edema.volume | Baseline.Crebral.edema.volume | Hematoma.mean.density |
| GPR | Coronary.heart.disease | Time.to.baseline.CT | Time.to.baseline.CT | Baseline.Contusion.volume | Hematoma.density.SD | Baseline.Contusion.volume |
| LMR | Island.sign | Eosinophil | GPR | Hematoma.density.SD | Baseline.Contusion.Surface.area | Hematoma.density.SD |
| GCS.score | Basophil | GPR | Island.sign | Hematoma.SR | Hematoma.SR | Hematoma.DCV |
| Baseline.Contusion.volume | Widening.and.deepening.of.cerebral.sulcus | GCS.score | Coronary.heart.disease | SIRI | Baseline.Contusion.volume | LMR |
| Hematoma.density.SD | Shallow.fissure.fission.in.the.cerebral.sulcus | White.blood.cell | GCS.score | Baseline.Contusion.Surface.area | Blood.glucose | GCS.score |
| Time.to.baseline.CT | SDH | Gender | SDH | GPR | GPR | Hematoma.SR |
| SIRI | Intraventricular.compression | SIRI | Temperature | uric.acid | uric.acid | SIRI |
| White.blood.cell | Eosinophil | Hematoma.density.SD | Eosinophil | Platelet | Platelet | MLR |

**Table S3** Ranking of All Variable Importances

| **Variable** | **Frequency** |
| --- | --- |
| Baseline.Contusion.volume | 5 |
| Hematoma.density.SD | 5 |
| Hematoma.DCV | 5 |
| GPR | 5 |
| Hematoma.SR | 4 |
| GCS.score | 4 |
| Eosinophil | 4 |
| Time.to.baseline.CT | 3 |
| Hematoma.mean.density | 3 |
| LMR | 3 |
| SDH | 2 |
| Island.sign | 2 |
| Baseline.Contusion.Surface.area | 2 |
| Coronary.heart.disease | 2 |
| White.blood.cell | 2 |
| Platelet | 2 |
| uric.acid | 2 |
| Gender | 1 |
| Intraventricular.compression | 1 |
| Widening.and.deepening.of.cerebral.sulcus | 1 |
| Shallow.fissure.fission.in.the.cerebral.sulcus | 1 |
| Temperature | 1 |
| Basophil | 1 |
| Blood.glucose | 1 |
| y | 0 |
| Age | 0 |
| coutusion.Site | 0 |
| SAH | 0 |
| EDH | 0 |
| Skull.Fracture | 0 |
| Midline.shift | 0 |
| Black.hole.sign | 0 |
| Blend.sign | 0 |
| Satellite.sign | 0 |
| Baseline.Crebral.edema.volume | 0 |
| Hypertension | 0 |
| Diabetes | 0 |
| Heart.rate | 0 |
| Systolic.blood.pressure | 0 |
| Diastolic.blood.pressure | 0 |
| Neutrophil | 0 |
| Lymphocyte | 0 |
| Monocyte | 0 |
| Red.blood.cell | 0 |
| Hemoglobin | 0 |
| RDW | 0 |
| Blood.Potassium | 0 |
| Blood.sodium | 0 |
| urea | 0 |
| Creatinine | 0 |
| Total.bilirubin | 0 |
| Total.protein | 0 |
| Albumin | 0 |
| AST | 0 |
| ALT | 0 |
| PT | 0 |
| INR | 0 |
| APTT | 0 |
| FIB | 0 |
| TT | 0 |
| D.dimer | 0 |
| SII | 0 |
| NLR | 0 |
| SIRI | 0 |
| PLR | 0 |
| DFR | 0 |
| MLR | 0 |
| RPR | 0 |
| PNI | 0 |
| APRI | 0 |
| uHG | 0 |
| Anterior.horns.of.the.bilateral.lateral.ventricles | 0 |
| Mean.arterial.pressure | 0 |
| Ultra.early.edema.expansion.rate | 0 |

**Figure S3** Spearman correlation heatmap of features associated with HP prediction in frontal lobe contusion. In this heatmap, red indicates positive correlation, blue indicates negative correlation, and darker colors represent larger absolute values of correlation.

**Table S4** Key parameters of the Support Vector Machines (SVM) model

| **Parameter Name** | **Value** |
| --- | --- |
| cost | 5 |
| gamma | 0.01 |
| seed | 123 |

**Table S5** Key parameters of the K Nearest Neighbors (KNN) model

| **Parameter Name** | **Value** |
| --- | --- |
| k  seed | 13  123 |

**Table S6** Key parameters of the Decision Trees model

| **Parameter Name** | **Value** |
| --- | --- |
| leaf_nodes | 22 |
| split_nodes | 21 |
| seed | 123 |

**Table S7** Key parameters of the Artificial Neural Networks (ANNs) model

| **Parameter Name** | **Value** |
| --- | --- |
| bestTune | 3 |
| size decay | 0.1 |
| seed | 123 |

**Table S8** Key parameters of the Naive Bayes model

| **Parameter Name** | **Value** |
| --- | --- |
| adjust | 0.8 |
| laplace | 0 |
| seed | 123 |

**Table S9** Key parameters of the Random Forest model

| **Parameter Name** |  | **Value** |
| --- | --- | --- |
| ntree |  | 500 |
| nodesize |  | 10 |
| seed |  | 123 |

**Table S10** Key parameters of the Boosted Regression Tree (BRT) model

| **Parameter Name** | **Value** |
| --- | --- |
| best_iter | 2569 |
| seed | 123 |

**Table S11** Key parameters of the Light Gradient Boosting Machine (LightGBM) model

| **Parameter Name** | **Value** |
| --- | --- |
| num_leaves | 15 |
| learning_rate | 0.05 |
| min_child_samples  colsample_bytree  subsample  verbose  nrounds  seed | 5  0.8  0.8  -1  200  123 |

**Table S12** Key parameters of the Logistic Regression model

| **Parameter Name** | **Value** |
| --- | --- |
| C | 1 |

**Table S13** Key parameters of the eXtreme Gradient Boosting (XGBoost) model

| **Parameter Name** | **Value** |
| --- | --- |
| eta | 0.1 |
| gamma | 0.25 |
| max_depth | 3 |
| subsample | 0.8 |
| colsample_bytree | 0.6 |
| nrounds | 100 |
| min_child_weight | 3 |
| max_delta_step | 3 |
| seed | 2345 |

**Table S14**  Full performance metrics of all candidate machine learning models in internal test set and external validation cohort

| Model | | auc | | auc95% | | | auc95% | | | best cut | | | Accuracy | | | Sensitivity/Recall | | | | Precision | | | | F1 score | | | Specificity | | | | |  |  |  |  |
| --- | --- | --- | --- | --- | --- | --- | --- | --- | --- | --- | --- | --- | --- | --- | --- | --- | --- | --- | --- | --- | --- | --- | --- | --- | --- | --- | --- | --- | --- | --- | --- | --- | --- | --- | --- |
| xgb | | 0.764 | | 0.696 | | | 0.831 | | | 0.315 | | | 0.736 | | | 0.696 | | | | 0.533 | | | | 0.604 | | | 0.753 | | | | |  |  |  |  |
| logistic | | 0.754 | | 0.684 | | | 0.824 | | | 0.332 | | | 0.757 | | | 0.623 | | | | 0.573 | | | | 0.597 | | | 0.812 | | | | |  |  |  |  |
| lightGBM | | 0.72 | | 0.649 | | | 0.791 | | | 0.201 | | | 0.690 | | | 0.725 | | | | 0.476 | | | | 0.575 | | | 0.676 | | | | |  |  |  |  |
| brt | | 0.747 | | 0.679 | | | 0.816 | | | 0.253 | | | 0.653 | | | 0.812 | | | | 0.444 | | | | 0.574 | | | 0.588 | | | | |  |  |  |  |
| rf | | 0.726 | | 0.655 | | | 0.797 | | | 0.228 | | | 0.603 | | | 0.87 | | | | 0.411 | | | | 0.558 | | | 0.494 | | | | |  |  |  |  |
| nb | | 0.674 | | 0.594 | | | 0.753 | | | 0.199 | | | 0.716 | | | 0.565 | | | | 0.506 | | | | 0.534 | | | 0.776 | | | | |  |  |  |  |
| ANNs | | 0.745 | | 0.675 | | | 0.815 | | | 0.353 | | | 0.745 | | | 0.696 | | | | 0.545 | | | | 0.611 | | | 0.765 | | | | |  |  |  |  |
| KNN | | 0.658 | | 0.579 | | | 0.737 | | | 0.423 | | | 0.707 | | | 0.449 | | | | 0.492 | | | | 0.47 | | | 0.812 | | | | |  |  |  |  |
| SVM | | 0.801 | | 0.742 | | | 0.859 | | | 0.237 | | | 0.665 | | | 0.899 | | | | 0.459 | | | | 0.608 | | | 0.571 | | | | |  |  |  |  |
| DT | | 0.65 | | 0.576 | | | 0.725 | | | 0.155 | | | 0.594 | | | 0.754 | | | | 0.394 | | | | 0.517 | | | 0.529 | | | | |  |  |  |  |
| **Data_Source** | **Model** | **AUC** | **AUC_CI_Lower** | | **AUC_CI_Upper** | **Best_Threshold** | | **Accuracy** | **Accuracy_CI_Lower** | | **Accuracy_CI_Upper** | **Sensitivity** | | **Sensitivity_CI_Lower** | **Sensitivity_CI_Upper** | | **Specificity** | **Specificity_CI_Lower** | **Specificity_CI_Upper** | | **Precision** | **Precision_CI_Lower** | **Precision_CI_Upper** | | **F1_Score** | **F1_CI_Lower** | | **F1_CI_Upper** | **HL_Chisq** | **HL_df** | **HL_pvalue** | | **Calibration_Slope** | **Calibration_Intercept** | **Brier_Score** |
| Test | XGBoost | 0.7635 | 0.6964 | | 0.8306 | 0.3146 | | 0.7364 | 0.6757 | | 0.7911 | 0.6957 | | 0.4251 | 0.6393 | | 0.7529 | 0.7927 | 0.9106 | | 0.5333 | 0.5731 | 0.8008 | | 0.6038 | 0.5075 | | 0.6909 | 5.6164 | 8 | 0.6901 | | 0.8317 | -0.112 | 0.170358 |
| Test | Logistic Regression | 0.7539 | 0.6836 | | 0.8242 | 0.3322 | | 0.7573 | 0.6979 | | 0.8103 | 0.6232 | | 0.4538 | 0.6869 | | 0.8118 | 0.7764 | 0.8937 | | 0.5733 | 0.4983 | 0.7371 | | 0.5972 | 0.4963 | | 0.6914 | 8.3758 | 8 | 0.3976 | | 0.8538 | -0.029 | 0.172675 |
| Test | LightGBM | 0.7202 | 0.6489 | | 0.7915 | 0.2009 | | 0.6904 | 0.6276 | | 0.7484 | 0.7246 | | 0.3778 | 0.5759 | | 0.6765 | 0.7875 | 0.9124 | | 0.4762 | 0.6038 | 0.8254 | | 0.5747 | 0.481 | | 0.6628 | 60.6714 | 8 | 0 | | 0.4243 | 0.1569 | 0.19486 |
| Test | BRT | 0.7471 | 0.6788 | | 0.8155 | 0.2528 | | 0.6527 | 0.5887 | | 0.7129 | 0.8116 | | 0.356 | 0.5356 | | 0.5882 | 0.8113 | 0.9373 | | 0.4444 | 0.6994 | 0.8957 | | 0.5744 | 0.0753 | | 0.2128 | 3.5798 | 8 | 0.8929 | | 1.0349 | -0.146 | 0.174114 |
| Test | Random Forest | 0.7263 | 0.6554 | | 0.7973 | 0.228 | | 0.6025 | 0.5374 | | 0.665 | 0.8696 | | 0.3303 | 0.4953 | | 0.4941 | 0.8242 | 0.9548 | | 0.411 | 0.7668 | 0.9386 | | 0.5581 | 0.0503 | | 0.1808 | 21.6752 | 8 | 0.0056 | | 0.7213 | -0.203 | 0.181449 |
| Test | Naive Bayes | 0.6736 | 0.5937 | | 0.7534 | 0.1986 | | 0.7155 | 0.6538 | | 0.7718 | 0.5652 | | 0.39 | 0.6224 | | 0.7765 | 0.7463 | 0.8714 | | 0.5065 | 0.4404 | 0.6842 | | 0.5342 | 0.4317 | | 0.6275 | 399.5679 | 8 | 0 | | 0.1207 | 0.4135 | 0.207207 |
| Test | ANNs | 0.7451 | 0.6751 | | 0.8151 | 0.3527 | | 0.7448 | 0.6846 | | 0.7988 | 0.6957 | | 0.4358 | 0.652 | | 0.7647 | 0.7953 | 0.9118 | | 0.5455 | 0.5731 | 0.8008 | | 0.6115 | 0.5128 | | 0.7018 | 11.9126 | 8 | 0.1551 | | 0.5968 | -0.17 | 0.178416 |
| Test | Decision Tree | 0.6504 | 0.5763 | | 0.7246 | 0.1551 | | 0.5941 | 0.5289 | | 0.657 | 0.7536 | | 0.3101 | 0.4827 | | 0.5294 | 0.7579 | 0.9046 | | 0.3939 | 0.6351 | 0.8495 | | 0.5174 | 0.1176 | | 0.27 | 74.5137 | 7 | 0 | | 0.2902 | -0.376 | 0.238018 |
| Test | KNN | 0.6581 | 0.5794 | | 0.7368 | 0.4231 | | 0.7071 | 0.645 | | 0.764 | 0.4493 | | 0.3638 | 0.6211 | | 0.8118 | 0.7159 | 0.8424 | | 0.4921 | 0.3292 | 0.5738 | | 0.4697 | 0.3622 | | 0.5714 | 16.8786 | 6 | 0.0097 | | 0.557 | -0.063 | 0.192617 |
| Test | SVM | 0.8005 | 0.7416 | | 0.8594 | 0.2369 | | 0.6653 | 0.6016 | | 0.7248 | 0.8986 | | 0.3732 | 0.5471 | | 0.5706 | 0.8662 | 0.9725 | | 0.4593 | 0.8021 | 0.9582 | | 0.6078 | 0.5217 | | 0.686 | 17.5911 | 8 | 0.0245 | | 1.2702 | -0.074 | 0.16817 |
| External validation | SVM(External validation) | 0.7239 | 0.6766 | | 0.7713 | 0.2369 | | 0.6907 | 0.6454 | | 0.7335 | 0.7253 | | 0.5346 | 0.668 | | 0.6667 | 0.7165 | 0.8296 | | 0.6027 | 0.6543 | 0.7887 | | 0.6584 | 0.6709 | | 0.7615 | 76.6378 | 8 | 0 | | 0.9487 | 0.6723 | 0.23802 |

|  | XGBoost | Logistic Regression | LightGBM | BRT | Random Forest | NaïveBayes | ANNS | Decision Tree | KNN | SVM |
| --- | --- | --- | --- | --- | --- | --- | --- | --- | --- | --- |
| XGBoost | 1 | 0.846 | 0.386 | 0.738 | 0.456 | 0.092 | 0.71 | 0.027 | 0.046 | 0.417 |
| Logistic Regression | 0.846 | 1 | 0.51 | 0.854 | 0.589 | 0.14 | 0.862 | 0.048 | 0.076 | 0.319 |
| LightGBM | 0.386 | 0.51 | 1 | 0.593 | 0.905 | 0.394 | 0.626 | 0.038 | 0.253 | 0.089 |
| BRT | 0.738 | 0.854 | 0.593 | 1 | 0.679 | 0.171 | 0.967 | 0.061 | 0.095 | 0.247 |
| Random Forest | 0.456 | 0.589 | 0.905 | 0.679 | 1 | 0.184 | 0.559 | 0.148 | 0.057 | 0.009 |
| Naïve Bayes | 0.092 | 0.14 | 0.394 | 0.171 | 0.184 | 1 | 0.079 | 0.677 | 0.674 | 0 |
| ANNS | 0.71 | 0.862 | 0.626 | 0.967 | 0.559 | 0.079 | 1 | 0.07 | 0.027 | 0.076 |
| Decision Tree | 0.027 | 0.048 | 0.038 | 0.061 | 0.148 | 0.677 | 0.07 | 1 | 0.889 | 0.002 |
| KNN | 0.046 | 0.076 | 0.253 | 0.095 | 0.057 | 0.674 | 0.027 | 0.889 | 1 | 0 |
| SVM | 0.417 | 0.319 | 0.089 | 0.247 | 0.009 | 0 | 0.076 | 0.002 | 0 | 1 |

**Table S15** The DeLong test compares results across the models

**Table S16 Completed TRIPOD+AI checklist for the present study**

**说明：D=仅模型开发研究；E=仅外部验证研究；D;E=开发+验证均适用；Reported(Y/N/NA)：Y=完整报告；N=缺失；NA=本条不适用于本研究；Location：标注正文对应段落/图表/补充表，审稿人可直接跳转核查**

**| Section & Item No. | D/E Scope | TRIPOD+AI Checklist Item Full Text | Reported (Y/N/NA) | Location (Manuscript Page/Section/Figure/Table) | Brief Explanation if N/NA |**

**| **Title** | | | | |**

**| 1 | D;E | Identify the study as developing or evaluating a multivariable prediction model, target population, and predicted outcome | Y | Title page | Manuscript title clearly states multimodal ML model for frontal lobe contusion DHP prediction |**

**| **Abstract** | | | | |**

**| 2 | D;E | Follow TRIPOD+AI for Abstract 13-item checklist | Y | Abstract | Abstract revised to separate internal (AUC=0.801) and external (AUC=0.724) results with all core metrics |**

**| **Introduction** | | | | |**

**| 3a | D;E | Explain clinical background, diagnostic/prognostic context, rationale, existing prediction models | Y | Introduction 1st–3rd para | Summarized prior TBI models and research gap for frontal-specific DHP prediction |**

**| 3b | D;E | Target population, model intended use, end users (clinicians) | Y | Introduction last para | Target: adult frontal contusion patients in ED; tool for neurosurgeons’ risk stratification |**

**| 3c | D;E | Describe known demographic health inequalities | Y | Discussion Limitations | Noted limitation of only Chinese tertiary hospital cohorts |**

**| 4 | D;E | Clear study objectives (model development + independent external validation) | Y | Introduction final paragraph | Two primary aims: build interpretable multimodal SVM; cross-region external validation |**

**| **Methods – Data & Participants** | | | | |**

**| 5a | D;E | Data source, cohort type, representativeness (development vs external dataset separately) | Y | 2.1 Research Objects | Dev: Ningxia tertiary hospital 2020.8–2025.10; External: Hunan tertiary hospital 2020–2025, cross-geographic independent cohort |**

**| 5b | D;E | Participant recruitment start/end dates, follow-up time window | Y | 2.1 | Enrollment dates clearly stated; follow-up CT restricted within 72 h post injury |**

**| 6a | D;E | Study setting, number & location of clinical centers | Y | 2.1 | Two geographically separated Chinese tertiary neurosurgery centers |**

**| 6b | D;E | Full inclusion and exclusion criteria | Y | 2.1, Figure 2 Flowchart | All age, GCS, CT time, surgery, incomplete data criteria listed clearly |**

**| 6c | D;E | Relevant treatments received during study period | NA | — | No intervention/treatment variables used as predictors; NA |**

**| 7 | D;E | All data preprocessing, quality control, harmonization across subgroups | Y | 2.2 Statistical Analysis | Z-score standardization, binary 0/1 coding, exclusion of patients with missing core data |**

**| 8a | D;E | Full outcome definition, time horizon, measurement method & rationale | Y | 2.1 DHP definition | DHP = hematoma volume increase ≥25% on 72h follow-up CT; intermediate 10%–25% group excluded + sensitivity analysis added |**

**| 8b | D;E | Qualifications of personnel assessing outcome (radiologists) | Y | 2.1 Imaging measurement | Two radiologists ≥5y experience blinded to clinical data; third senior expert for discrepancy adjudication |**

**| 8c | D;E | Blinding of outcome assessors to clinical predictors | Y | 2.1 | Radiologists blinded to admission lab/clinical information |**

**| 9a | D | Source of initial candidate predictors (literature, clinical routine) | Y | 2.3.1 Feature screening | 74 multimodal predictors from routine admission CT, lab, clinical indices based on TBI pathophysiology |**

**| 9b | D;E | Full definition of all final predictors, measurement timing | Y | Supplementary Table S1 | All 10 core predictors fully defined with plain English descriptions |**

**| 9c | D;E | Qualifications of personnel measuring imaging predictors | Y | 2.1 | Same blinded radiologist team for all CT volumetric measurements |**

**| 10 | D;E | Sample size justification + formal sample size calculation | Y | 2.2 Sample size subsection | Two methods (EPV rule + Riley Cox-Snell formula), minimum events threshold met |**

**| 11 | D;E | Missing data handling, exclusion rules for incomplete cases | Y | 2.2, Supplementary Table S2 | All patients with incomplete core data excluded at enrollment; no post-hoc imputation required |**

**| **Methods – Analytical Workflow** | | | | |**

**| 12a | D | Data splitting strategy (train/internal test, cross-validation) | Y | 2.1, 2.3.2 | Stratified 7:3 split; 5-fold grid search cross-validation for hyperparameter tuning |**

**| 12b | D | Variable transformation, rescaling, functional forms | Y | 2.2 | Continuous features standardized via training-set mean/SD only |**

**| 12c | D | Model type, rationale, full building pipeline, hyperparameter tuning, internal validation | Y | 2.3.1 & 2.3.2 | 7 consensus feature-selection methods; 10 ML algorithms, full hyperparameter grid listed in Supplementary S4–S13 |**

**| 12d | D;E | Handling clustering/heterogeneity across centers | Y | Discussion Limitations | Two independent hospital cohorts analyzed separately; cross-center performance compared |**

**| 12e | D;E | All performance metrics (discrimination, calibration, clinical utility) with rationale | Y | 2.2, 3.2–3.5 | AUROC, sensitivity, specificity, F1, calibration curve, Brier, HL test, DCA all reported |**

**| 12f | E | Any model recalibration/updating in external cohort | NA | — | No model recalibration performed on external data; NA |**

**| 12g | E | Method to calculate predicted risk in external cohort | Y | 2.3.2 | Fixed SVM model trained solely on development training set applied to external raw features |**

**| 13 | D;E | Class imbalance adjustment methods + rationale if used | Y | 2.2 | No oversampling/weighting applied; sensitivity analysis with SMOTE/weighted SVM added for comparison |**

**| 14 | D;E | Fairness assessment across demographic subgroups | N | Discussion | Future research proposed for subgroup fairness analysis; clearly noted limitation |**

**| 15 | D | Model output format (probability), threshold selection rules | Y | 3.2 | Continuous predicted risk probabilities; no fixed clinical threshold proposed |**

**| 16 | D;E | Differences between development vs external dataset (setting, eligibility, predictors) | Y | 2.1, Table 1 | Cohort baseline characteristics compared; geographic difference clarified |**

**| 17 | D;E | Ethics committee name, approval number, informed consent waiver | Y | 2.1 Ethics paragraph | Ethics No. KYLL-2025-2410; consent waived for anonymized retrospective data |**

**| **Open Science Section** | | | | |**

**| 18a | D;E | Funding source, funder role in study design/analysis | Y | Manuscript Declarations | Natural Science Foundation of Ningxia; funders had no role in research conduct |**

**| 18b | D;E | All authors conflict of interest statement | Y | Declarations | All authors declare no competing interests |**

**| 18c | D;E | Study protocol availability statement | N | Discussion | No pre-registered study protocol; explained as single-center retrospective retrospective cohort |**

**| 18d | D;E | Study registration information | N | Discussion | Retrospective observational study without clinical trial registration |**

**| 18e | D;E | Raw data access statement | Y | Declarations | De-identified data available from corresponding author on reasonable formal request |**

**| 18f | D;E | Analysis code availability statement | N | Discussion | R code not publicly deposited; can be provided to reviewers upon request |**

**| 19 | D;E | Patient & public involvement (PPI) during research | NA | — | Study was retrospective secondary data analysis without PPI activities |**

**| **Results** | | | | |**

**| 20a | D;E | Participant flowchart, number of outcome/non-outcome cases | Y | Figure 2 CONSORT-style flowchart | Clear participant screening flow for both development & external cohorts |**

**| 20b | D;E | Baseline table with demographics, predictors, missing volume | Y | Supplementary Table S22 | Baseline characteristics of dev & external cohorts side-by-side |**

**| 20c | E | Predictor distribution comparison dev vs external cohort | Y | Supplementary Table S22 | Inter-cohort variable distribution comparison provided |**

**| 21 | D;E | Number of participants/events for training, tuning, test, external analysis | Y | 2.1, Table 1 | Training n=560, internal test n=240, external n=442 clearly listed |**

**| 22 | D | Full predictive model specification | N | Discussion | SVM kernel model cannot be simplified to fixed formula; SHAP interpretation used instead |**

**| 23a | D;E | All performance metrics with 95% CIs for internal & external cohorts | Y | Table 1, Supplementary S14 | AUC, sensitivity, specificity, F1 all supplemented with confidence intervals |**

**| 23b | D;E | Cross-center performance heterogeneity results | Y | 3.2 | Internal AUC=0.801, external AUC=0.724, attenuation explained |**

**| 24 | E | Results of any model recalibration/updating | NA | — | No model updating performed in external validation |**

**| **Discussion** | | | |**

**| 25 | D;E | Overall result interpretation, fairness discussion | Y | Discussion main text | Interpret model performance alongside demographic limitations |**

**| 26 | D;E | Full study limitations (sample, overfitting, generalizability, bias) | Y | Discussion Limitations subsection | Retrospective design, single-country tertiary hospitals, lack prospective validation clearly stated |**

**| 27a | D | Handling missing/poor-quality predictors during clinical use | Y | Conclusion & Discussion | All predictors are routine admission tests with minimal missingness in clinical practice |**

**| 27b | D | End-user expertise required to implement model | Y | Discussion | Neurosurgeons/emergency physicians without ML expertise can apply bedside |**

**| 27c | D;E | Future validation & implementation research directions | Y | Limitations final paragraph | Prospective multi-regional multi-hospital external validation proposed |**

**Fig. S4** Shapley additive explanation plots, applicable to patients with frontal lobe contusion hematoma progression and non-progression. **A.** A patient with hematoma progression in sample 1. **B.** A patient without hematoma progression in sample2.

**Fig.S5. ROC curves of the SVM model in sensitivity analysis incorporating patients with intermediate hematoma expansion (10%–25% volume increase)**

**Fig.S6. ROC curve of the SVM model constructed using features with consensus selection frequency ≥ 1**
